# Development and Validation of a Prognostic Classification Model Predicting Postoperative Adverse Outcomes in Older Surgical Patients Using a Machine Learning Algorithm: Retrospective Observational Network Study

**DOI:** 10.2196/42259

**Published:** 2023-11-13

**Authors:** Jung-Yeon Choi, Sooyoung Yoo, Wongeun Song, Seok Kim, Hyunyoung Baek, Jun Suh Lee, Yoo-Seok Yoon, Seonghae Yoon, Hae-Young Lee, Kwang-il Kim

**Affiliations:** 1 Departmentof Internal Medicine Seoul National University Bundang Hospital Seongnam-si Republic of Korea; 2 Office of eHealth Research and Business Seoul National University Bundang Hospital Seongnam-si Republic of Korea; 3 Department of Health Science and Technology, Graduate School of Convergence Science and Technology Seoul National University Seongnam-si Republic of Korea; 4 Department of Surgery G Sam Hospital Gunpo Republic of Korea; 5 Department of Surgery Seoul National University Bundang Hospital Seongnam-si Republic of Korea; 6 Department of Surgery Seoul National University College of Medicine Seoul Republic of Korea; 7 Department of Clinical Pharmacology and Therapeutic Seoul National University Bundang Hospital Seongnam-si Republic of Korea; 8 Department of Internal Medicine Seoul National University Hospital Seoul Republic of Korea; 9 Department of Internal Medicine Seoul National University College of Medicine Seoul Republic of Korea

**Keywords:** CDM, common data model, patient-level prediction, OHDSI, Observational Health Data Sciences and Informatics, postoperative outcome, postoperative, surgery, elderly, elder, predict, adverse event, adverse outcome, geriatric, older adult, ageing, model, algorithm

## Abstract

**Background:**

Older adults are at an increased risk of postoperative morbidity. Numerous risk stratification tools exist, but effort and manpower are required.

**Objective:**

This study aimed to develop a predictive model of postoperative adverse outcomes in older patients following general surgery with an open-source, patient-level prediction from the Observational Health Data Sciences and Informatics for internal and external validation.

**Methods:**

We used the Observational Medical Outcomes Partnership common data model and machine learning algorithms. The primary outcome was a composite of 90-day postoperative all-cause mortality and emergency department visits. Secondary outcomes were postoperative delirium, prolonged postoperative stay (≥75th percentile), and prolonged hospital stay (≥21 days). An 80% versus 20% split of the data from the Seoul National University Bundang Hospital (SNUBH) and Seoul National University Hospital (SNUH) common data model was used for model training and testing versus external validation. Model performance was evaluated using the area under the receiver operating characteristic curve (AUC) with a 95% CI.

**Results:**

Data from 27,197 (SNUBH) and 32,857 (SNUH) patients were analyzed. Compared to the random forest, Adaboost, and decision tree models, the least absolute shrinkage and selection operator logistic regression model showed good internal discriminative accuracy (internal AUC 0.723, 95% CI 0.701-0.744) and transportability (external AUC 0.703, 95% CI 0.692-0.714) for the primary outcome. The model also possessed good internal and external AUCs for postoperative delirium (internal AUC 0.754, 95% CI 0.713-0.794; external AUC 0.750, 95% CI 0.727-0.772), prolonged postoperative stay (internal AUC 0.813, 95% CI 0.800-0.825; external AUC 0.747, 95% CI 0.741-0.753), and prolonged hospital stay (internal AUC 0.770, 95% CI 0.749-0.792; external AUC 0.707, 95% CI 0.696-0.718). Compared with age or the Charlson comorbidity index, the model showed better prediction performance.

**Conclusions:**

The derived model shall assist clinicians and patients in understanding the individualized risks and benefits of surgery.

## Introduction

Owing to the shift in age demographics, the demand for surgical procedures in the aging population has increased. In 2007, more than one-third of all surgical procedures for inpatients were performed in the older population, which doubled by 2020 [1-3]. For older patients, pre-existing comorbidities or frailty increase the risk of morbidity or mortality after surgery. Moreover, surgical complications increase medical costs, unnecessary hospitalization, and functional dependency. Thus, changes in proper health care management should be undertaken [4,5].

The development of a risk prediction model for older surgical patients aims to quantify patients’ individualized risks and identify risk-benefit based on their comorbidities, pathophysiological characteristics, and additional evaluations. Since surgical risks increase with age, chronological age may not capture the wide range of health statuses of older patients [6]. Therefore, a number of intuitive, organ-based, and physiological vulnerability-based risk prediction models have been developed and validated in older patients undergoing surgery [7]. Prediction of adverse outcomes after surgery is essential for surgeons’ decision-making and patients’ expectations after surgery, particularly regarding the patients’ quality of life. However, additional evaluations of the physiological vulnerability of older patients may require time, space, and manpower.

The Observational Health Data Sciences and Informatics (OHDSI), which originated from the Observational Medical Outcomes Partnership (OMOP), was developed to properly use observational health care databases. The OHDSI is an open collaborative network of international researchers who focus on methodological research, open-source analytics development, and clinical applications to improve the generation and dissemination of reliable medical evidence from observational data [8]. To unify medical terms or clinical coding in diverse electronic health record (EHR) environments worldwide, the OMOP common data model (CDM) was developed to standardize health care data [9].

We aimed to use a large-scale analytics approach to develop a prediction model for a patient’s individual risk of adverse outcomes of surgical procedures for those aged 65 years or older based on real-world observational EHR data. We also investigated the developed prognostic classification model across the OHDSI data network by validating it on external CDM data.

## Methods

### Sources of Data

Data for development and internal validation were collected from longitudinal observational EHR data collected between April 2003 and December 2020 from Seoul National University Bundang Hospital (SNUBH) and externally validated with EHR data from Seoul National University Hospital (SNUH) collected between October 2004 and December 2020. SNUBH and SNUH are university-affiliated tertiary hospitals located in the Seoul metropolitan area and the capital, respectively. Both hospitals offer inpatient, outpatient, and emergency department (ED) services. EHR data collected from both hospitals included the patients’ demographics, diagnoses, drug prescriptions (for outpatients), drug administrations (for inpatients and patients in the ED), operations, vital signs, and laboratory test results. The type of data sets captured in the 2 hospitals were similar. In this study, EHR data from each institution were converted to the OMOP CDM and used in the OHDSI’s large-scale data analytics framework. The OMOP CDM provides a homogenous format for health care data and standardization of underlying clinical coding systems, which enables sharing of the analysis code across participating data sets in the OHDSI data network [10].

### Ethical Considerations

Since the data sources were de-identified, the study protocol was reviewed and approved with a waiver of informed consent by the institutional review boards (IRBs) of each participating site which included the Seoul National University Bundang Hospital (X-1912-652-904) and Seoul National University Hospital (E-2011-013-1169). Our study strictly adheres to the guideline about machine learning predictive models in biomedical research [11].

### Study Design and Target Cohort

Our retrospective study target cohort consisted of all surgical inpatients aged >65 years who underwent general surgery at the Department of Surgery within 14 days of admission. We developed 4 types of prognostic models for predicting postoperative delirium, prolonged postoperative stay, composite outcome of all-cause mortality and ED visits, and prolonged hospital stay from admission to discharge using SNUBH CDM data.

To predict postoperative delirium, prolonged postoperative stay, and the composite outcome, the prediction time (t=0) and time-at-risk start date of the prediction window were set as the surgery date. The time-at-risk end date for postoperative delirium and prolonged postoperative stay prediction was set as the discharge date, and the time-at-risk end date for the composite outcome was defined as 90 days after surgery.

For the prediction of prolonged hospital stay, the prediction time and time-at-risk start date were set as the admission date and the time-at-risk end date was the discharge date.

**Figure 1:**
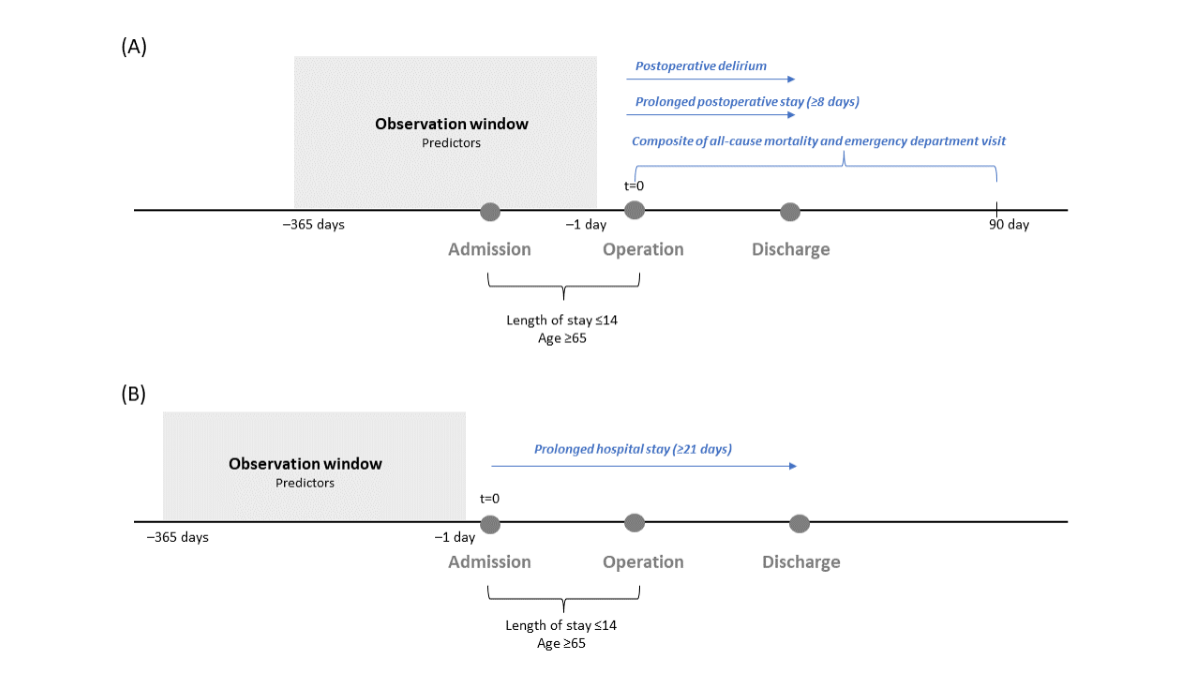
Study design for predicting postoperative outcomes in older patients. (A) Prediction of postoperative delirium, prolonged postoperative stay, and a composite of all-cause mortality and emergency department visits on the operation date. (B) Prediction of prolonged hospital stay on the admission date.

The predictor observation time window was defined as –365 days to –1 day relative to the time-at-risk start date for all 4 outcomes. We excluded patients for whom the observation period was less than 1 year. Figure 1 shows the study design for predicting different postoperative outcomes.

### Outcomes

The primary endpoint was a composite of all-cause mortality and ED visits up to 90 days after the operation date. For the ED visits, we only included patients who were admitted to the ED for over 24 hours. In previous studies, postoperative complications were usually reported within a period of 30 to 90 days after surgery [12,13]; in this study, a 90-day time-at-risk was used in consideration of the number of outcomes required to learn the model. The secondary outcomes were postoperative delirium, prolonged postoperative stay ≥75th percentile [13-15] (ie, ≥8 days in the SNUBH data set), and prolonged hospital stay of ≥21 days. Postoperative delirium was operationally defined as the initiation of antipsychotic medications (eg, haloperidol, quetiapine, or olanzapine) after surgery in patients who had not been prescribed these medications at any point during the entire observation period. Because the length of hospital stay is one of the main indicators of medical efficiency and hospital management, this study also defined prolonged hospital stay based on the data of hospitalization as a widely used performance indicator.

### Predictors

The covariates used in the predictive model included the following: demographics (sex and age), prior conditions (condition record and condition group era), number of distinct conditions, oral medications, procedures and observation (family history and medical history data), measurement value, number of hospital visits for different visit types, and the CHADS_2_-VASc (congestive heart failure, hypertension, age ≥75, diabetes, stroke [doubled], vascular disease, age 65-74, and sex category) score observed 365 days prior to the time-at-risk start date.

An additional analysis was performed using surgery information as a covariate. The prediction performance was evaluated when the surgery concept itself or surgical risk groups (intermediate- or high-risk surgery vs low-risk surgery) were added as predictors into the model. The surgical risk groups were categorized using a previously established risk-stratification model [15].

### Prediction Model Development and Validation

The standardized open-source OHDSI patient-level prediction package was used to develop and validate the model using observational health care data [16,17]. We developed a prognostic model using the SNUBH CDM development data set. The SNUBH CDM data set was split into training (80% of the data) and testing (20% of the data) sets to perform internal validation of the developed model. For 80% of the training data set, we used a 5-fold cross-validation for hyperparameter tuning and eventually obtained an optimal prediction model [18]. Using the OHDSI patient-level prediction framework, the least absolute shrinkage and selection operator (LASSO) logistic regression, gradient boosting machine, random forest, Adaboost, and decision tree models were developed in this study. The LASSO logistic regression model was used to develop predictive models using regularized logistic regression with an L1 (LASSO) prior, which applies equal shrinkage to the coefficients and enables automatic feature selection and better prediction accuracy owing to its reduced variance [19]. In the hyperparameter grid search, the default setting of OHDSI patient-level prediction was used (see Multimedia Appendix 1 for detailed grid search settings).

To evaluate the models, model discrimination was assessed using the area under the receiver operating characteristic curve (AUC), and model calibration was assessed by inspecting a calibration plot. The generalizability of the model was evaluated by performing internal validation using SNUBH test data and external validation using SNUH data for the model trained with SNUBH CDM data.

## Results

### Study Population

This study included data from 27,197 and 32,857 patients from the SNUBH and SNUH databases, respectively. The baseline demographic and outcome characteristics of the target population in the 2 databases are presented in Table 1. The SNUBH and SNUH data sets had an average age of 72.94 (SD 5.48) years and 72.17 (SD 5.54) years, respectively, with men accounting for 54.3% (n=14,769) and 54.15% (n=17,792) of patients, respectively. A similar distribution was found for severity and length of stay. Postoperative delirium occurred at an incidence of 2.81% (n=764) and 1.63% (n=536) in the SNUBH and SNUH populations, respectively.

The number of people eligible for inclusion in the target population, the outcome count, and the number of people lost due to each inclusion step for developing the 4 prognostic models for the different outcomes at SNUBH are presented in Multimedia Appendix 2.

**Table 1:** Characteristics of patients older than 65 years who underwent general surgery within 14 days of admission to Seoul National University Bundang Hospital (SNUBH) and Seoul National University Hospital (SNUH).

Characteristic			SNUBH		SNUH
Visits, n			27,197		32,857
Unique patients, n			23,782		29,281
Age (years), mean (SD)			72.94 (5.48)		72.17 (5.54)
**Sex, n (%)**					
	Male	14,768 (54.3)		17,792 (54.15)	
	Female	12,429 (45.7)		15,065 (45.85)	
Hospitalizations, mean (SD)			1.14 (0.43)		1.12 (0.40)
CHADS_2_-VASc^a^ score, mean (SD)			2.16 (0.99)		2.06 (0.93)
Charlson comorbidity index, mean (SD)			2.80 (1.86)		2.79 (1.80)
**Length of stay (days)**					
	Mean (SD)	11.86 (13.24)		12.86 (24.16)	
	Median (IQR)	9 (5-14)		9 (6-14)	
**Postoperative delirium during hospitalization**					
	Patients with postoperative delirium, n (%)	764 (2.81)		536 (1.63)	
	Days to delirium after surgery, mean (SD)	8.78 (13.90)		11.85 (17.61)	
	Days to delirium after surgery, median (IQR)	4 (2-10)		6 (3-14)	
**Top 5 most common surgeries at SNUBH, n (%)**					
	Laparoscopic cholecystectomy	3063 (11.26)		N/A^b^	
	Distal subtotal gastrectomy	1774 (6.52)		N/A	
	Colonoscopic procedure	1384 (5.09)		N/A	
	Exploratory laparotomy	1295 (4.76)		N/A	
	Lumpectomy of breast	863 (3.17)		N/A	
**Top 5 most common surgery at SNUH, n (%)**					
	Laparoscopic cholecystectomy	N/A		2462 (7.49)	
	Exploratory laparotomy	N/A		2056 (6.26)	
	Total thyroidectomy	N/A		1467 (4.46)	
	Pylorus-sparing Whipple operation	N/A		1277 (3.89)	
	Simple mastectomy	N/A		1069 (3.25)	

^a^CHADS_2_-VASc: congestive heart failure, hypertension, age ≥75, diabetes, stroke (doubled), vascular disease, age 65-74, and sex category.

^b^N/A: not applicable.

### Outcomes

The overall discriminative performance of the different prognostic models for the 2 data sets is described in Table 2. The calibration of the different models is presented in Multimedia Appendices 3-6 for each adverse outcome. The primary outcome of composite outcome of all-cause mortality and ED visits occurred in 11.4% (n=2800) and 6.7% (n=2120) of the patients from SNUBH and SNUH, respectively; postoperative delirium occurred in 2.8% (n=764) and 1.6% (n=536) of patients, respectively; prolonged postoperative stay occurred in 24.1% (n=6563) and 23.8% (n=7826) of patients, respectively; and prolonged hospital stay occurred in 6.8% (n=1853) and 6.4% (n=2102) of patients, respectively.

**Figure 2:**
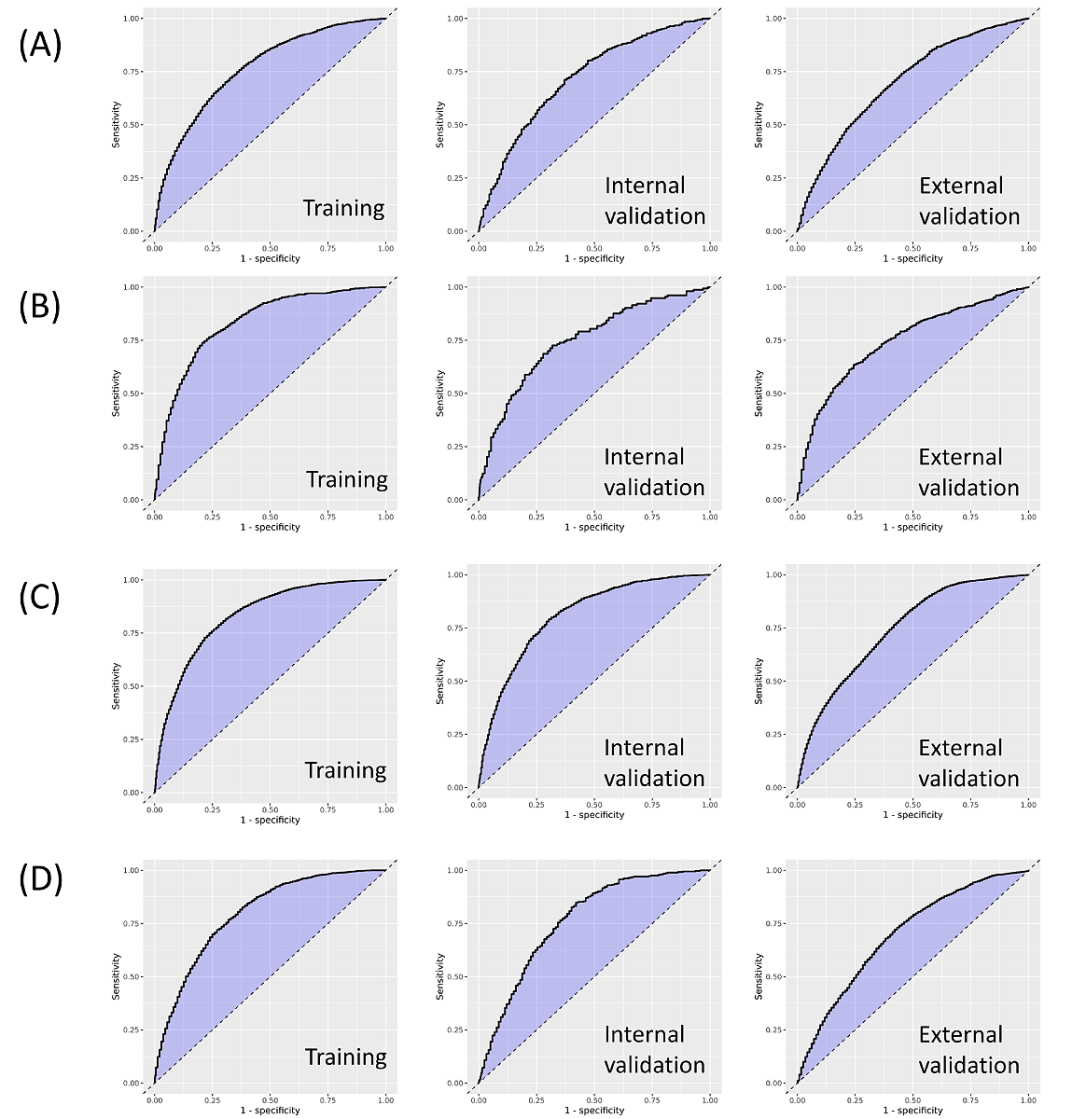
Receiver operating characteristic plots of least absolute shrinkage and selection operator logistic regression of the SNUBH training, SNUBH test (interval validation), and SNUH test (external validation) data sets for the adverse outcomes of (A) composite outcome of all-cause mortality and emergency department visits, (B) postoperative delirium, (C) prolonged postoperative stay, and (D) prolonged hospital stay. The y-axis is specificity and the x-axis is 1-specificity. SNUBH: Seoul National University Bundang Hospital; SNUH: Seoul National University Hospital.

**Figure 3:**
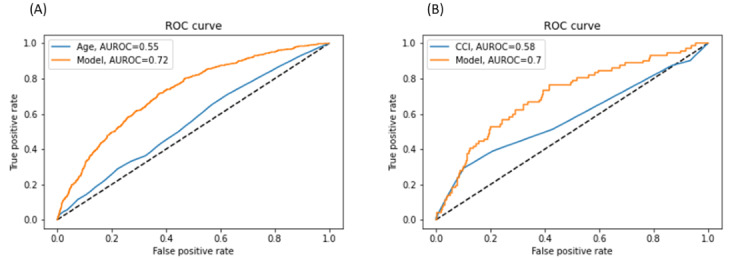
Comparison of primary outcome prediction between our model of least absolute shrinkage and selection operator logistic regression and (A) age or (B) CCI. AUROC: area under the receiver operating characteristic curve; CCI: Charlson comorbidity index; ROC: receiver operating characteristic.

Among the 5 predictive models, LASSO logistic regression generally showed the best overall performance in internal and external validation across all adverse outcomes. The receiver operating characteristic curve of LASSO logistic regression across internal and external validations is presented in Figure 2. For the primary composite outcome, the AUCs (95% CI) of the model were 0.723 (0.701-0.744) and 0.703 (0.692-0.714) for internal validation and external validation, respectively. When we compared the age or Charlson comorbidity index, the LASSO logistic regression model showed better performance in predicting a composite outcome of all-cause mortality and ED visits (Figure 3). For postoperative delirium outcomes, the AUCs of the model were 0.754 (0.713-0.794) for SNUBH data and 0.754 (0.727-0.772) for SNUH data. For prolonged postoperative stay, the AUCs of the model were 0.813 (0.800-0.825) for SNUBH data and 0.747 (0.741-0.753) for SNUH data. Regarding prolonged hospital stay, the AUCs of the model were 0.770 (0.749-0.792) for SNUBH data and 0.707 (0.696-0.718) for SNUH data. In all 4 outcomes, the random forest model showed the highest performance with an AUC of 0.950-0.987 in training; however, it was found that the performance was lowered to 0.677-0.708 in external validation.

When surgery information was added as a predictor, overall AUC performance improved for all outcomes. Table 3 shows the performance of the LASSO logistic regression model when surgery predictors were added. The highest performance was observed when the surgery concept itself was added as a predictor. In particular, the performance was improved when the surgery concepts were included for the prolonged postoperative and hospital stays.

For the primary outcome of the composite outcome, of the 14,361 candidate predictors in the SNUBH data set, 315 (2.2%) predictors were selected through LASSO regression. The covariates selected for the predictive model and their covariate values are presented in Multimedia Appendix 7. The 5 negative predictors with the strongest association with the primary outcome were measurement values of albumin, prothrombin time, PR interval, condition of “inguinal hernia,” and drug of azelastine. The 3 positive predictors with the strongest association with the primary outcome were ED visit count during the observation period, measurement values of segmented neutrophils per 100 leukocytes in the blood, and the measurement value of glucose.

To predict postoperative delirium, the final model included 141 of 14,687 (1.0%) candidate predictors (Multimedia Appendix 8). The 3 strongest positive predictors were age, condition era group of “malignant tumor of esophagus,” and the measurement value of glucose.

For the prediction of prolonged postoperative stay, 465 of 14,687 (3.2%) candidate predictors were chosen (Multimedia Appendix 9). Measurement values of albumin, condition of “inguinal hernia,” and procedure of “CT of thyroid with contrast” were negatively associated with prolonged postoperative stay, while condition of “malignant tumor of esophagus” and procedure of “plain film of head” were positively associated.

In the predictive model that analyzed prolonged hospital stay, 237 of 14,181 (1.7%) candidate predictors were selected (Multimedia Appendix 10). Measurement values of albumin, condition of “hernia of abdominal wall,” and “neoplasm of breast” were negatively associated with prolonged hospital stay, while measurement values of total bilirubin, C reactive protein, and condition of osteitis were positively associated.

**Table 2:** Internal and external validations of the 4 prognostic models for different outcomes.

Outcome				SNUBH^a^				SNUH^b^
				Training (n=21,766)		Test (n=5431)		External validation (n=32,857)
**Composite outcome of all-cause mortality and emergency department visits**								
	Sample size meeting inclusion criteria, n (%)			19,602 (90.06)		4938 (90.92)		31,658 (96.35)
	Outcome, n (%)			2224 (11.35)		576 (11.66)		2120 (6.7)
	**AUROC^c^ (95% CI)**							
		LASSO^d^ logistic regression	0.770 (0.760-0.781)		0.723 (0.701-0.744)		0.703 (0.692-0.714)	
		Gradient boosting machine	0.832 (0.822-0.841)		0.733 (0.712-0.755)		0.652 (0.639-0.664)	
		AdaBoost	0.757 (0.747-0.767)		0.712 (0.689-0.734)		0.695 (0.684-0.707)	
		Random forest	0.971 (0.965-0.976)		0.742 (0.721-0.764)		0.698 (0.686-0.71)	
		Decision tree	0.784 (0.773-0.794)		0.662 (0.637-0.686)		0.593 (0.581-0.606)	
**Postoperative delirium**								
	Sample size meeting inclusion criteria, n (%)			21,766 (100)		5431 (100)		32,857 (100)
	Outcome, n (%)			611 (2.81)		153 (2.82)		536 (1.63)
	**AUROC (95% CI)**							
		LASSO logistic regression	0.837 (0.822-0.852)		0.754 (0.713-0.794)		0.750 (0.727-0.772)	
		Gradient boosting machine	0.922 (0.911-0.934)		0.771 (0.732-0.809)		0.699 (0.678-0.720)	
		AdaBoost	0.849 (0.837-0.861)		0.742 (0.702-0.782)		0.703 (0.678-0.727)	
		Random forest	0.950 (0.938-0.961)		0.744 (0.706-0.782)		0.677 (0.654-0.700)	
		Decision tree	0.826 (0.806-0.845)		0.616 (0.565-0.667)		0.644 (0.620-0.669)	
**Prolonged postoperative stay**								
	Sample size meeting inclusion criteria, n (%)			21,767 (100)		5430 (100)		32,857 (100)
	Outcome, n (%)			5254 (24.14)		1309 (24.11)		7826 (23.82)
	**AUROC (95% CI)**							
		LASSO logistic regression	0.835 (0.829-0.841)		0.813 (0.800-0.825)		0.747 (0.741-0.753)	
		Gradient boosting machine	0.989 (0.988-0.991)		0.820 (0.808-0.832)		0.721 (0.715-0.727)	
		AdaBoost	0.808 (0.802-0.814)		0.796 (0.783-0.810)		0.743 (0.737-0.749)	
		Random forest	0.987 (0.986-0.989)		0.814 (0.801-0.827)		0.708 (0.702-0.714)	
		Decision tree	0.842 (0.836-0.848)		0.736 (0.72-0.752)		0.623 (0.616-0.630)	
**Prolonged hospital stay**								
	Sample size meeting inclusion criteria, n (%)			21,755 (100)		5442 (100)		32,857 (100)
	Outcome, n (%)			1482 (6.81)		371 (6.82)		2102 (6.4)
	**AUROC (95% CI)**							
		LASSO logistic regression	0.801 (0.791-0.812)		0.770 (0.749-0.792)		0.707 (0.696-0.718)	
		Gradient boosting machine	0.910 (0.903-0.918)		0.762 (0.740-0.783)		0.712 (0.700-0.723)	
		AdaBoost	0.795 (0.785-0.805)		0.743 (0.719-0.766)		0.708 (0.697-0.719)	
		Random forest	0.958 (0.953-0.964)		0.753 (0.730-0.777)		0.707 (0.696-0.718)	
		Decision tree	0.798 (0.786-0.810)		0.694 (0.666-0.722)		0.628 (0.616-0.640)	

^a^SNUBH: Seoul National University Bundang Hospital.

^b^SNUH: Seoul National University Hospital.

^c^AUROC: area under the receiver operating characteristic curve.

^d^LASSO: least absolute shrinkage and selection operator.

**Table 3:** Internal and external validations of the least absolute shrinkage and selection operator logistic regression model after adding a surgery variable to the predictor.

Outcome, AUROC^a^ (95% CI)		SNUBH^b^		SNUH^c^
		Training (n=21,766)	Test (n=5431)	External validation (n=32,857)
**Composite outcome of all-cause mortality and emergency department visits**				
	With no use of surgery	0.770 (0.706-0.781)	0.723 (0.701-0.744)	0.703 (0.692-0.714)
	With surgery concepts	0.776 (0.765-0.786)	0.739 (0.718-0.760)	0.712 (0.700-0.723)
	With surgery risk type^d^	0.764 (0.754-0.774)	0.731 (0.71-0.751)	0.703 (0.692-0.714)
**Postoperative delirium**				
	With no use of surgery	0.837 (0.822-0.852)	0.754 (0.713-0.794)	0.75 (0.727-0.772)
	With surgery concepts	0.859 (0.845-0.873)	0.764 (0.726-0.803)	0.759 (0.737-0.781)
	With surgery risk type	0.824 (0.809-0.839)	0.744 (0.705-0.783)	0.76 (0.739-0.781)
**Prolonged postoperative stay**				
	With no use of surgery	0.835 (0.829-0.841)	0.813 (0.800-0.825)	0.747 (0.741-0.753)
	With surgery concepts	0.870 (0.865-0.875)	0.840 (0.828-0.851)	0.776 (0.771-0.782)
	With surgery risk type	0.819 (0.813-0.826)	0.794 (0.781-0.807)	0.756 (0.75-0.762)
**Prolonged hospital stay**				
	With no use of surgery	0.801 (0.791-0.812)	0.770 (0.749-0.792)	0.707 (0.696-0.718)
	With surgery concepts	0.867 (0.858-0.875)	0.830 (0.81-0.850)	0.756 (0.744-0.767)
	With surgery risk type	0.771 (0.760-0.781)	0.741 (0.721-0.762)	0.726 (0.717-0.736)

^a^AUROC: area under the receiver operating characteristic curve.

^b^SNUBH: Seoul National University Bundang Hospital.

^c^SNUH: Seoul National University Hospital.

^d^The surgery risk type was classified into two types: (1) intermediate or high and (2) low.

## Discussion

### Principal Findings

This study demonstrated the feasibility of developing patient-level predictive models to identify adverse outcomes after surgery among older adults. The LASSO logistic regression model performed well, with an internal AUC (95% CI) of 0.723 (0.701-0.744), and also applied well to another data set with an external AUC of 0.703 (0.692-0.714) to predict a composite outcome of all-cause mortality and ED visits.

The covariates selected by our model included variables that correlated with the outcomes of older patients undergoing surgery in previous studies. In the patient-level prediction model for the 3 outcomes, a low serum albumin level was selected as the strongly correlated covariate. Low albumin levels were associated with postoperative mortality, ED visits, postoperative delirium, and prolonged hospital or postoperative stay. This is consistent with the results of previous studies. Low serum albumin concentration has a clear relationship with all-cause mortality in older patients and is a strong prognostic parameter for postoperative outcomes [20-23]. Covariates associated with postoperative delirium were also consistent with previous research on advanced age and operation-specific risk [24]. We also confirmed that surgical disease was the strongest risk factor for a prolonged hospital stay. Breast cancer or hernia presented with shorter hospital stays, and patients with pancreatobiliary-related disease (elevated bilirubin) and C reactive protein levels required a longer time to discharge.

### Strengths and Limitations

This study bears several strengths. We developed and validated our patient-level predictive model using 2 large data sets covering a wide variety of covariates, including demographics, medical conditions, and prior health behaviors, that were collected through routine care. As comprehensive evaluation among older patients undergoing surgery is necessary, additional effort is required for a comprehensive geriatric assessment. The prediction model using indicators collected during routine care was easier to implement.

Our developed model, presented in Table 2, did not include surgery type or surgical risk as a covariate. Although we observed some improvement when incorporating surgery concepts or surgical risk, as shown in Table 3, the difference in the composite outcome of all-cause mortality and ED visits was not significant. Surgeons can estimate the level of risk associated with a particular surgery based on their experience. Therefore, a risk stratification model that reflects patients’ characteristics is likely to be more useful to surgeons. Despite not including any information about surgery, our risk stratification model that accounts for patients’ characteristics may have significant practical value and can be applied widely in various hospitals regardless of the surgery type or risk.

However, this study also bears several limitations. First, since only CDM-based outcomes were measured, the mortality and ED visit rates may have been underdetected. Second, the incidence of delirium (1.6%-2.8%) may have been underestimated compared to previous results from a meta-analysis, as it was counted based on medication administration for symptom control [25]. Among patients older than 60-70 years, the incidence of postoperative delirium has been reported to be 10%-20% depending on the patient and surgical risk [26]. Furthermore, if delirium has occurred, the drug may not be used without risk of self-harm. Therefore, our operational definition of delirium could have underestimated its actual occurrence. The differences in delirium incidence between the 2 hospitals could be attributed to the differences in the clinical practice policies for delirium even though the patients’ ages and comorbidity statuses at baseline were similar between both hospitals. Third, there was a difference in the model prediction accuracy during external validation depending on the outcome. In our study, the external predictive value of the model for postoperative delirium, prolonged postoperative stay, and prolonged hospital stay was well-maintained; however, it was poor for a composite of mortality and ED revisits in the external validation. This is probably due to the hospital operation characteristics not being reflected in the model or missed outcomes rather than the patient characteristics since there was a difference in the outcome ratios of the 2 data sets (11.4% and 6.7% for SNUBH and SNUH, respectively). Additionally, we could not analyze out-of-hospital mortality and reported a composite outcome within 90 days. The predicted 30-day or 90-day mortality was not presented as a result in this study because a meaningful model could not be developed; such a model required the outcomes of more than 1000 patients [27], which our data set lacked. The composite outcome within 30 days also failed to show an AUC of 0.7 or higher in the internal and external validation; therefore, future studies with larger data sets are required.

### Conclusion

We developed and evaluated various patient-level predictions based on routinely collected EHR data that can identify older patients with higher risks of adverse outcomes after surgery. This was successfully developed from observational and OMOP CDM data and was easily transported to different institutions and externally validated. A next possible step may be to apply the model to EHRs, which can assist in decision-making, additional perioperative evaluation, and supportive care to prevent adverse outcomes after surgery.

## Appendix

Multimedia Appendix 1 Hyperparameter grid search settings.

Multimedia Appendix 2 Attrition diagram of target populations for developing 4 prognostic models of (A) a composite outcome of all-cause mortality and emergency department visits, (B) postoperative delirium, (C) prolonged postoperative stay, and (D) prolonged hospital stay.

Multimedia Appendix 3 Receiver operating characteristic (ROC) plots and calibration plots for the internal and external validations of different models predicting composite outcome of all-cause mortality and emergency department visit. In each validation result, the upper figure is the ROC plot and the lower figure is the calibration plot. The dotted line represents the perfect model calibration in the calibration plot.

Multimedia Appendix 4 Calibration plots for the internal and external validations of different models predicting postoperative delirium. In each validation result, the upper figure is the receiver operating characteristic curve plot and the lower figure is the calibration plot. The dotted line represents the perfect model calibration in the calibration plot.

Multimedia Appendix 5 Calibration plots for the internal and external validations of different models predicting prolonged stay. In each validation result, the upper figure is the receiver operating characteristic curve plot and the lower figure is the calibration plot. The dotted line represents the perfect model calibration in the calibration plot.

Multimedia Appendix 6 Calibration plots for the internal and external validations of different models predicting prolonged hospital stay. In each validation result, the upper figure is the receiver operating characteristic curve plot and the lower figure is the calibration plot. The dotted line represents the perfect model calibration in the calibration plot.

Multimedia Appendix 7 The 315 selected variables and covariate values of the least absolute shrinkage and selection operator logistic regression model for composite outcomes of all-cause mortality and emergency department visits 90 days after surgery.

Multimedia Appendix 8 The 141 selected variables and covariate values of the least absolute shrinkage and selection operator logistic regression model for postoperative delirium during hospitalization.

Multimedia Appendix 9 The 465 selected variables and covariate values of the least absolute shrinkage and selection operator logistic regression model for prolonged postoperative stay.

Multimedia Appendix 10 The 237 selected variables and covariate values of the least absolute shrinkage and selection operator logistic regression model for prolonged hospital stay.

## Abbreviations

AUC: area under the receiver operating characteristic curve
CDM: common data model
CHADS2-VASc: congestive heart failure, hypertension, age ≥75, diabetes, stroke (doubled), vascular disease, age 65-74, and sex category
ED: emergency department
EHR: electronic health record
IRB: institutional review board
LASSO: least absolute shrinkage and selection operator
OHDSI: Observational Health Data Sciences and Informatics
OMOP: Observational Medical Outcomes Partnership
SNUBH: Seoul National University Bundang Hospital
SNUH: Seoul National University Hospital

## Data Availability

The source code are provided on Github [28].
