# Supplementary material for: Development and Validation of a Prognostic Classification Model Predicting Postoperative Adverse Outcomes in Older Surgical Patients Using a Machine Learning Algorithm: Retrospective Observational Network Study
Source: J Med Internet Res. 2023 Nov 13;25:e42259. doi: 10.2196/42259 (PMC10682929; doi:10.2196/42259)
Supplement: Multimedia Appendix 1 [file jmir_v25i1e42259_app1.docx]

Hyper-parameter grid search settings

| **Parameter** | **Description** | **Values** |
| --- | --- | --- |
| **Gradient Boosting Machines** | | |
| earlyStopRound | Stopping after rounds without improvement | 25 |
| learningRate | The boosting learn rate | 0.005,0.01,0.1 |
| maxDepth | Max levels in a tree | 4,6,17 |
| minRows | Min data points in a node | 2 |
| ntrees | Number of trees | 1, 100, 1000 |
| Random Forest |  |  |
| maxDepth | Max levels in a tree | 4,10,17 |
| mtries | Number of features in each tree | square root of total features,5,20 |
| ntrees | Number of trees | 500 |
| **AdaBoost** | | |
| nEstimators | The maximum number of estimators at which boosting is terminated | 4 |
| learningRate | Learning rate shrinks the contribution of each classifier by learning_rate. There is a trade-off between learningRate and nEstimators | 1 |
| **Decision Tree** | | |
| lassWeight | “Balance” or “None” | None |
| maxDepth | The maximum depth of the tree | 10 |
| minImpuritySplit | Threshold for early stopping in tree growth. A node will split if its impurity is above the threshold, otherwise it is a leaf | 10^-7 |
| minSamplesLeaf | The minimum number of samples per leaf | 10 |
| minSamplesSplit | The minimum samples per split | 2 |
| **Random Forest** | | |
| maxDepth | Max levels in a tree | 4,10,17 |
| mtries | Number of features in each tree | -1 = square root of total features,5,20 |
| ntrees | Number of trees | 500 |
