# Supplementary material for: Development and Validation of a Prognostic Classification Model Predicting Postoperative Adverse Outcomes in Older Surgical Patients Using a Machine Learning Algorithm: Retrospective Observational Network Study
Source: J Med Internet Res. 2023 Nov 13;25:e42259. doi: 10.2196/42259 (PMC10682929; doi:10.2196/42259)
Supplement: Multimedia Appendix 2 [file jmir_v25i1e42259_app2.docx]

Attrition diagram of target populations for developing four prognostic models of (A) composite outcome of all-cause mortality and emergency department visit (B) postoperative delirium (C) prolonged postoperative stay, and (D) prolonged hospital stay
