# Supplementary material for: Development and Validation of a Prognostic Classification Model Predicting Postoperative Adverse Outcomes in Older Surgical Patients Using a Machine Learning Algorithm: Retrospective Observational Network Study
Source: J Med Internet Res. 2023 Nov 13;25:e42259. doi: 10.2196/42259 (PMC10682929; doi:10.2196/42259)
Supplement: Multimedia Appendix 4 [file jmir_v25i1e42259_app4.docx]

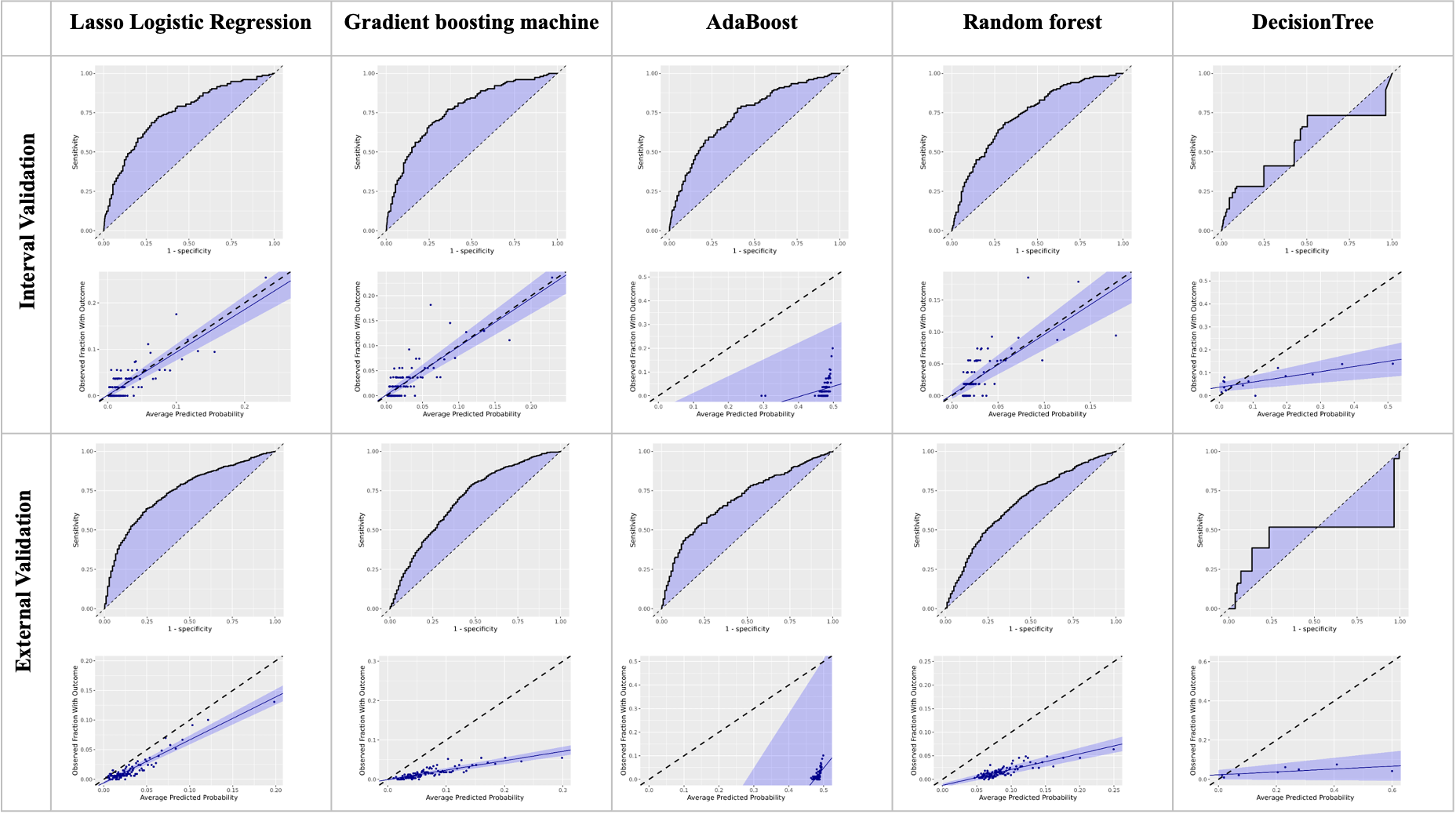


Calibration plots for the internal and external validations of different models predicting postoperative delirium. In each validation result, the upper figure is the ROC plot and the lower figure is the calibration plot. The dotted line represents the perfect model calibration in the calibration plot.
