# Supplementary material for: Development and Validation of a Prognostic Classification Model Predicting Postoperative Adverse Outcomes in Older Surgical Patients Using a Machine Learning Algorithm: Retrospective Observational Network Study
Source: J Med Internet Res. 2023 Nov 13;25:e42259. doi: 10.2196/42259 (PMC10682929; doi:10.2196/42259)
Supplement: Multimedia Appendix 7 [file jmir_v25i1e42259_app7.docx]

Selected 315 variables and covariate values of the Lasso Logistic Regression model for composite outcomes of all-cause mortality and emergency department visits 90 days after surgery

| **covariateId** | **covariateName** | **covariateValue** |
| --- | --- | --- |
| 9203923 | visit_occurrence concept count during day -365 through -1 concept_count relative to index: Emergency Room Visit | 2.467140008 |
| 3.01261E+12 | measurement value during day -365 through -1 days relative to index: Segmented neutrophils/100 leukocytes in Blood by Automated count (percent) | 1.092979473 |
| 3.01405E+12 | measurement value during day -365 through -1 days relative to index: Glucose [Mass/volume] in Blood by Test strip manual (Unknown unit) | 0.749306533 |
| 3.02702E+12 | measurement value during day -365 through -1 days relative to index: Heart rate (Non-specific) | 0.748237224 |
| 4304943502 | procedure_occurrence during day -365 through -1 days relative to index: Endoscopic retrograde cholangiopancreatography | 0.729718893 |
| 1383815 | drug_era only per oral during day -365 through -1 days relative to index: isosorbide | 0.653166471 |
| 3.01368E+12 | measurement value during day -365 through -1 days relative to index: Urea nitrogen [Mass/volume] in Serum or Plasma (milligram per deciliter) | 0.607424983 |
| 4163971502 | procedure_occurrence during day -365 through -1 days relative to index: Laparoscopic cholecystectomy | 0.484025652 |
| 4180793102 | condition_occurrence during day -365 through -1 days relative to index: Malignant tumor of pancreas | 0.452412163 |
| 4299435802 | observation during day -365 through -1 days relative to index: Site of distant metastasis | 0.444534492 |
| 196151210 | condition_era group during day -365 through -1 days relative to index: Functional disorder of intestine | 0.41355783 |
| 4.08916E+12 | measurement value during day -365 through -1 days relative to index: Grip strength (kilogram) | 0.403487038 |
| 1716903 | drug_era only per oral during day -365 through -1 days relative to index: moxifloxacin | 0.388334382 |
| 4181187210 | condition_era group during day -365 through -1 days relative to index: Inflammatory disorder of head | 0.368961785 |
| 19005626 | drug_era only per oral during day -365 through -1 days relative to index: trimetazidine | 0.365780947 |
| 441561102 | condition_occurrence during day -365 through -1 days relative to index: Low tension glaucoma | 0.363200264 |
| 256451210 | condition_era group during day -365 through -1 days relative to index: Bronchitis | 0.330062627 |
| 3.00318E+12 | measurement value during day -365 through -1 days relative to index: Sodium [Moles/volume] in Urine (millimole per liter) | 0.328297373 |
| 199754102 | condition_occurrence during day -365 through -1 days relative to index: Primary malignant neoplasm of pancreas | 0.294889913 |
| 4028244210 | condition_era group during day -365 through -1 days relative to index: Chronic disease of cardiovascular system | 0.293697887 |
| 19025198 | drug_era only per oral during day -365 through -1 days relative to index: pinaverium | 0.291185547 |
| 1328165 | drug_era only per oral during day -365 through -1 days relative to index: diltiazem | 0.285615681 |
| 192671102 | condition_occurrence during day -365 through -1 days relative to index: Gastrointestinal hemorrhage | 0.283610494 |
| 432571102 | condition_occurrence during day -365 through -1 days relative to index: Malignant lymphoma | 0.282696601 |
| 4028265210 | condition_era group during day -365 through -1 days relative to index: Infectious disease of cardiovascular system | 0.276836109 |
| 4202832502 | procedure_occurrence during day -365 through -1 days relative to index: Intubation | 0.275433062 |
| 4068261210 | condition_era group during day -365 through -1 days relative to index: Bile duct proliferation | 0.27038051 |
| 4332246210 | condition_era group during day -365 through -1 days relative to index: Aneurysm | 0.263741988 |
| 4130829502 | procedure_occurrence during day -365 through -1 days relative to index: Platelet transfusion | 0.261102042 |
| 4099350210 | condition_era group during day -365 through -1 days relative to index: Infectious disease of genitourinary system | 0.25724525 |
| 4115107210 | condition_era group during day -365 through -1 days relative to index: Mass of female genital structure | 0.254810531 |
| 4181343102 | condition_occurrence during day -365 through -1 days relative to index: Malignant tumor of esophagus | 0.247696534 |
| 3.0119E+12 | measurement value during day -365 through -1 days relative to index: Phosphate [Mass/volume] in Serum or Plasma (milligram per deciliter) | 0.247220623 |
| 198124210 | condition_era group during day -365 through -1 days relative to index: Kidney disease | 0.245994961 |
| 1703603 | drug_era only per oral during day -365 through -1 days relative to index: famciclovir | 0.220695049 |
| 40481633102 | condition_occurrence during day -365 through -1 days relative to index: Azotemia | 0.215288054 |
| 3.0276E+12 | measurement value during day -365 through -1 days relative to index: Mean blood pressure (millimeter mercury column) | 0.210576473 |
| 4116238210 | condition_era group during day -365 through -1 days relative to index: Malignant tumor of digestive organ | 0.208295436 |
| 1904 | CHADS2VASc | 0.20669665 |
| 37018657102 | condition_occurrence during day -365 through -1 days relative to index: Primary adenocarcinoma of gallbladder | 0.206579218 |
| 19066992 | drug_era only per oral during day -365 through -1 days relative to index: silymarin | 0.206000098 |
| 797399 | drug_era only per oral during day -365 through -1 days relative to index: gabapentin | 0.189434843 |
| 916005 | drug_era only per oral during day -365 through -1 days relative to index: solifenacin | 0.183111429 |
| 372924102 | condition_occurrence during day -365 through -1 days relative to index: Cerebral artery occlusion | 0.181547783 |
| 4183452210 | condition_era group during day -365 through -1 days relative to index: Otitis | 0.181445738 |
| 443392210 | condition_era group during day -365 through -1 days relative to index: Malignant neoplastic disease | 0.18122002 |
| 19011339 | drug_era only per oral during day -365 through -1 days relative to index: mosapride | 0.181093284 |
| 4183041102 | condition_occurrence during day -365 through -1 days relative to index: Abdominal discomfort | 0.180385294 |
| 43009053 | drug_era only per oral during day -365 through -1 days relative to index: itopride hydrochloride | 0.177222138 |
| 4083787210 | condition_era group during day -365 through -1 days relative to index: Skin or mucosa lesion | 0.174299042 |
| 906780 | drug_era only per oral during day -365 through -1 days relative to index: metoclopramide | 0.174254281 |
| 4182562210 | condition_era group during day -365 through -1 days relative to index: Lower abdominal pain | 0.173858782 |
| 4177244210 | condition_era group during day -365 through -1 days relative to index: Malignant tumor of female genital organ | 0.171652641 |
| 4174764102 | condition_occurrence during day -365 through -1 days relative to index: Late gastric cancer | 0.164623272 |
| 1110410 | drug_era only per oral during day -365 through -1 days relative to index: morphine | 0.164114659 |
| 321052210 | condition_era group during day -365 through -1 days relative to index: Peripheral vascular disease | 0.163214641 |
| 43008986 | drug_era only per oral during day -365 through -1 days relative to index: Lactobacillus helveticus R0052 | 0.162874711 |
| 1361711 | drug_era only per oral during day -365 through -1 days relative to index: nitroglycerin | 0.159756811 |
| 4189640210 | condition_era group during day -365 through -1 days relative to index: Neoplasm of hematopoietic cell type | 0.15386562 |
| 134736210 | condition_era group during day -365 through -1 days relative to index: Backache | 0.14956226 |
| 1516766 | drug_era only per oral during day -365 through -1 days relative to index: repaglinide | 0.145840074 |
| 443387102 | condition_occurrence during day -365 through -1 days relative to index: Malignant tumor of stomach | 0.142159588 |
| 1125315 | drug_era only per oral during day -365 through -1 days relative to index: acetaminophen | 0.142012313 |
| 2.001E+15 | measurement value during day -365 through -1 days relative to index: RF211_right Total T-score (score) | 0.141159523 |
| 4116964210 | condition_era group during day -365 through -1 days relative to index: Mass of musculoskeletal structure | 0.139184449 |
| 4306292210 | condition_era group during day -365 through -1 days relative to index: Upper abdominal pain | 0.13672519 |
| 1036228 | drug_era only per oral during day -365 through -1 days relative to index: sucralfate | 0.134086593 |
| 4324448502 | procedure_occurrence during day -365 through -1 days relative to index: CT of pancreas with contrast | 0.131633789 |
| 2E+15 | measurement value during day -365 through -1 days relative to index: Indicator of Surgery Cancer (score) | 0.130625817 |
| 4304092502 | procedure_occurrence during day -365 through -1 days relative to index: CT of abdomen and pelvis | 0.129592611 |
| 993631 | drug_era only per oral during day -365 through -1 days relative to index: magnesium oxide | 0.129214365 |
| 1796458 | drug_era only per oral during day -365 through -1 days relative to index: cefdinir | 0.127231931 |
| 967861 | drug_era only per oral during day -365 through -1 days relative to index: magnesium citrate | 0.124598033 |
| 4181345210 | condition_era group during day -365 through -1 days relative to index: Malignant tumor of biliary tract | 0.119608159 |
| 3.01641E+12 | measurement value during day -365 through -1 days relative to index: Fibrinogen [Mass/volume] in Platelet poor plasma by Coagulation assay (milligram per deciliter) | 0.118411069 |
| 4143249802 | observation during day -365 through -1 days relative to index: Determination of acuity level | 0.117582719 |
| 4304358502 | procedure_occurrence during day -365 through -1 days relative to index: Diagnostic procedure | 0.114953335 |
| 46272861802 | observation during day -365 through -1 days relative to index: Radiotherapy to thorax | 0.114799208 |
| 1713332 | drug_era only per oral during day -365 through -1 days relative to index: amoxicillin | 0.113319729 |
| 19049105 | drug_era only per oral during day -365 through -1 days relative to index: potassium chloride | 0.113199595 |
| 252662210 | condition_era group during day -365 through -1 days relative to index: Tracheobronchial disorder | 0.113129889 |
| 4287399210 | condition_era group during day -365 through -1 days relative to index: Retroperitoneal mass | 0.1058153 |
| 1353766 | drug_era only per oral during day -365 through -1 days relative to index: propranolol | 0.105729696 |
| 319835102 | condition_occurrence during day -365 through -1 days relative to index: Congestive heart failure | 0.104605029 |
| 35604767 | drug_era only per oral during day -365 through -1 days relative to index: methylephedrine | 0.103501509 |
| 4110575102 | condition_occurrence during day -365 through -1 days relative to index: Adenocarcinoma of rectum | 0.103036724 |
| 4131770210 | condition_era group during day -365 through -1 days relative to index: Neoplasm of central nervous system | 0.102906831 |
| 4022173502 | procedure_occurrence during day -365 through -1 days relative to index: Transfusion of red blood cells | 0.102073799 |
| 3654996210 | condition_era group during day -365 through -1 days relative to index: Peripheral arterial disease | 0.100539913 |
| 1503297 | drug_era only per oral during day -365 through -1 days relative to index: metformin | 0.098951133 |
| 4300595210 | condition_era group during day -365 through -1 days relative to index: Neoplasm of endocrine system | 0.09834851 |
| 19011331 | drug_era only per oral during day -365 through -1 days relative to index: barium sulfate | 0.098254408 |
| 4335825502 | procedure_occurrence during day -365 through -1 days relative to index: Transthoracic echocardiography | 0.097277153 |
| 761012210 | condition_era group during day -365 through -1 days relative to index: Lesion of vertebra | 0.096002132 |
| 4227449102 | condition_occurrence during day -365 through -1 days relative to index: Spondylosis | 0.093817167 |
| 437113102 | condition_occurrence during day -365 through -1 days relative to index: Asthenia | 0.093039973 |
| 4338120210 | condition_era group during day -365 through -1 days relative to index: Altered bowel function | 0.09162994 |
| 1580747 | drug_era only per oral during day -365 through -1 days relative to index: sitagliptin | 0.09124544 |
| 45763613210 | condition_era group during day -365 through -1 days relative to index: Colonic lesion | 0.090629725 |
| 316866210 | condition_era group during day -365 through -1 days relative to index: Hypertensive disorder | 0.090277001 |
| 193518102 | condition_occurrence during day -365 through -1 days relative to index: Intestinal obstruction | 0.089937525 |
| 3.01518E+12 | measurement value during day -365 through -1 days relative to index: Erythrocyte distribution width [Entitic volume] by Automated count (femtoliter) | 0.088971322 |
| 4022171502 | procedure_occurrence during day -365 through -1 days relative to index: Transfusion of fresh frozen plasma | 0.088803 |
| 4181062210 | condition_era group during day -365 through -1 days relative to index: Inflammation of skin and/or subcutaneous tissue | 0.087674892 |
| 4044391210 | condition_era group during day -365 through -1 days relative to index: Neuropathy due to diabetes mellitus | 0.086990199 |
| 4214956802 | observation during day -365 through -1 days relative to index: History of clinical finding in subject | 0.085596819 |
| 36879181 | drug_era only per oral during day -365 through -1 days relative to index: streptococcus faecalis | 0.083300478 |
| 1510813 | drug_era only per oral during day -365 through -1 days relative to index: rosuvastatin | 0.082886711 |
| 1350310 | drug_era only per oral during day -365 through -1 days relative to index: cilostazol | 0.082208897 |
| 35610218802 | observation during day -365 through -1 days relative to index: APACHE II (Acute Physiology and Chronic Health Evaluation II) score | 0.080938291 |
| 43009008 | drug_era only per oral during day -365 through -1 days relative to index: rebamipide | 0.080426526 |
| 4311115210 | condition_era group during day -365 through -1 days relative to index: Intracranial mass | 0.079453022 |
| 1550557 | drug_era only per oral during day -365 through -1 days relative to index: prednisolone | 0.079214814 |
| 4208660210 | condition_era group during day -365 through -1 days relative to index: Cholangiocarcinoma of biliary tract | 0.073978925 |
| 1707164 | drug_era only per oral during day -365 through -1 days relative to index: metronidazole | 0.07251182 |
| 904453 | drug_era only per oral during day -365 through -1 days relative to index: esomeprazole | 0.070205388 |
| 199866102 | condition_occurrence during day -365 through -1 days relative to index: Acute gastritis | 0.065999003 |
| 19088167 | drug_era only per oral during day -365 through -1 days relative to index: ambroxol | 0.065236612 |
| 4111798210 | condition_era group during day -365 through -1 days relative to index: Neoplasm of digestive organ | 0.064472221 |
| 3.02736E+12 | measurement value during day -365 through -1 days relative to index: T wave axis (Unknown unit) | 0.063701645 |
| 1318853 | drug_era only per oral during day -365 through -1 days relative to index: nifedipine | 0.063254705 |
| 36715927102 | condition_occurrence during day -365 through -1 days relative to index: Primary cholangiocarcinoma of intrahepatic biliary tract | 0.058654348 |
| 43021974210 | condition_era group during day -365 through -1 days relative to index: Complication associated with device | 0.058614332 |
| 3.03512E+12 | measurement value during day -365 through -1 days relative to index: Erythrocytes [#/area] in Urine sediment by Microscopy high power field (per high power field) | 0.057305029 |
| 312437102 | condition_occurrence during day -365 through -1 days relative to index: Dyspnea | 0.057163252 |
| 4024561102 | condition_occurrence during day -365 through -1 days relative to index: Pain in lower limb | 0.056805742 |
| 953076 | drug_era only per oral during day -365 through -1 days relative to index: famotidine | 0.055929481 |
| 4294382502 | procedure_occurrence during day -365 through -1 days relative to index: Esophagogastroduodenoscopy | 0.053416403 |
| 1136422 | drug_era only per oral during day -365 through -1 days relative to index: levocetirizine | 0.051379211 |
| 4041664210 | condition_era group during day -365 through -1 days relative to index: Difficulty breathing | 0.050990488 |
| 317585210 | condition_era group during day -365 through -1 days relative to index: Aortic aneurysm | 0.048515716 |
| 956874 | drug_era only per oral during day -365 through -1 days relative to index: furosemide | 0.046781002 |
| 1189596 | drug_era only per oral during day -365 through -1 days relative to index: dihydrocodeine | 0.046665244 |
| 36878639 | drug_era only per oral during day -365 through -1 days relative to index: ivy leaf extract | 0.045917478 |
| 1396131 | drug_era only per oral during day -365 through -1 days relative to index: ferrous sulfate | 0.045000892 |
| 198091210 | condition_era group during day -365 through -1 days relative to index: Primary malignant neoplasm of retroperitoneum | 0.044846508 |
| 81902102 | condition_occurrence during day -365 through -1 days relative to index: Urinary tract infectious disease | 0.043114566 |
| 4176793210 | condition_era group during day -365 through -1 days relative to index: Congenital anomaly of abdomen | 0.04219382 |
| 37117305502 | procedure_occurrence during day -365 through -1 days relative to index: CT of abdomen and pelvis without contrast | 0.041171215 |
| 19112563 | drug_era only per oral during day -365 through -1 days relative to index: calcium polystyrene sulfonate product | 0.039320218 |
| 4132130210 | condition_era group during day -365 through -1 days relative to index: Dilatation of aorta | 0.035487124 |
| 734354 | drug_era only per oral during day -365 through -1 days relative to index: pregabalin | 0.035336405 |
| 4215685802 | observation during day -365 through -1 days relative to index: Past history of procedure | 0.035054938 |
| 19092433 | drug_era only per oral during day -365 through -1 days relative to index: ebastine | 0.032550842 |
| 40479625210 | condition_era group during day -365 through -1 days relative to index: Atherosclerosis of artery | 0.030783752 |
| 4058335502 | procedure_occurrence during day -365 through -1 days relative to index: CT of chest | 0.029938021 |
| 437312210 | condition_era group during day -365 through -1 days relative to index: Bleeding | 0.028884041 |
| 937439 | drug_era only per oral during day -365 through -1 days relative to index: bethanechol | 0.026056921 |
| 438878102 | condition_occurrence during day -365 through -1 days relative to index: Liver function tests abnormal | 0.025398915 |
| 948078 | drug_era only per oral during day -365 through -1 days relative to index: pantoprazole | 0.02482576 |
| 1337620 | drug_era only per oral during day -365 through -1 days relative to index: capecitabine | 0.024284893 |
| 37016775210 | condition_era group during day -365 through -1 days relative to index: Traumatic and/or non-traumatic injury of back | 0.023484134 |
| 375807210 | condition_era group during day -365 through -1 days relative to index: Degenerative disorder of eye | 0.021615399 |
| 40166571 | drug_era only per oral during day -365 through -1 days relative to index: bepotastine | 0.020194617 |
| 4088035802 | observation during day -365 through -1 days relative to index: Screening status | 0.017241465 |
| 3.03617E+12 | measurement value during day -365 through -1 days relative to index: Chloride [Moles/volume] in Cerebral spinal fluid (millimole per liter) | 0.01560338 |
| 1526475 | drug_era only per oral during day -365 through -1 days relative to index: ezetimibe | 0.015240835 |
| 912362 | drug_era only per oral during day -365 through -1 days relative to index: ammonium chloride | 0.014997239 |
| 440029210 | condition_era group during day -365 through -1 days relative to index: Viral disease | 0.014959448 |
| 317585102 | condition_occurrence during day -365 through -1 days relative to index: Aortic aneurysm | 0.01482501 |
| 4154630210 | condition_era group during day -365 through -1 days relative to index: Malignant neoplasm of genitourinary organ | 0.0135437 |
| 4159131102 | condition_occurrence during day -365 through -1 days relative to index: Dyslipidemia | 0.013426372 |
| 437663102 | condition_occurrence during day -365 through -1 days relative to index: Fever | 0.012800858 |
| 40481633210 | condition_era group during day -365 through -1 days relative to index: Azotemia | 0.012580114 |
| 42538572210 | condition_era group during day -365 through -1 days relative to index: Degeneration of spine | 0.01173627 |
| 739138 | drug_era only per oral during day -365 through -1 days relative to index: sertraline | 0.010106727 |
| 3.00208E+12 | measurement value during day -365 through -1 days relative to index: Tricuspid valve Maximum regurgitant blood flow velocity during systole by US.doppler (meter per second) | 0.008520488 |
| 4324347502 | procedure_occurrence during day -365 through -1 days relative to index: CT of liver with contrast | 0.008326016 |
| 4170143210 | condition_era group during day -365 through -1 days relative to index: Respiratory tract infection | 0.006843131 |
| 4164337210 | condition_era group during day -365 through -1 days relative to index: Polyp of large intestine | 0.00666408 |
| 437541210 | condition_era group during day -365 through -1 days relative to index: Glaucoma | 0.003742219 |
| 1192710 | drug_era only per oral during day -365 through -1 days relative to index: chlorpheniramine | 0.003301393 |
| 438878210 | condition_era group during day -365 through -1 days relative to index: Liver function tests abnormal | 0.002799534 |
| 4301699210 | condition_era group during day -365 through -1 days relative to index: Neuropathy | 0.001073845 |
| 1351557 | drug_era only per oral during day -365 through -1 days relative to index: candesartan | 0.000888053 |
| 441561210 | condition_era group during day -365 through -1 days relative to index: Low tension glaucoma | 0.000825881 |
| 43008987 | drug_era only per oral during day -365 through -1 days relative to index: Lactobacillus rhamnosus R0011 | 0.000752908 |
| 42898675 | drug_era only per oral during day -365 through -1 days relative to index: Bacillus subtilis | 0.000342596 |
| 319835210 | condition_era group during day -365 through -1 days relative to index: Congestive heart failure | 7.98E-05 |
| 4048027210 | condition_era group during day -365 through -1 days relative to index: Neuropathy associated with endocrine disorder | 2.18E-09 |
| 3.02104E+12 | measurement value during day -365 through -1 days relative to index: Iron binding capacity [Mass/volume] in Serum or Plasma (microgram per deciliter) | -6.11E-05 |
| 798874 | drug_era only per oral during day -365 through -1 days relative to index: clonazepam | -0.000827071 |
| 439708210 | condition_era group during day -365 through -1 days relative to index: Disorders of initiating and maintaining sleep | -0.001559356 |
| 435657210 | condition_era group during day -365 through -1 days relative to index: Dyssomnia | -0.002624219 |
| 974166 | drug_era only per oral during day -365 through -1 days relative to index: hydrochlorothiazide | -0.005750514 |
| 4289001210 | condition_era group during day -365 through -1 days relative to index: Acute gastroenteritis | -0.007809734 |
| 1322184 | drug_era only per oral during day -365 through -1 days relative to index: clopidogrel | -0.007817303 |
| 432661210 | condition_era group during day -365 through -1 days relative to index: Inflammatory disease of mucous membrane | -0.008622956 |
| 1139042 | drug_era only per oral during day -365 through -1 days relative to index: acetylcysteine | -0.009318434 |
| 36713361102 | condition_occurrence during day -365 through -1 days relative to index: Primary adenocarcinoma of ascending colon | -0.009968088 |
| 4308811210 | condition_era group during day -365 through -1 days relative to index: Neoplasm of soft tissue | -0.011312305 |
| 19136187 | drug_era only per oral during day -365 through -1 days relative to index: streptokinase | -0.012351737 |
| 317576210 | condition_era group during day -365 through -1 days relative to index: Coronary arteriosclerosis | -0.013013687 |
| 4134593210 | condition_era group during day -365 through -1 days relative to index: Chronic digestive system disorder | -0.013044756 |
| 443398210 | condition_era group during day -365 through -1 days relative to index: Malignant tumor of intestine | -0.014298492 |
| 40484102102 | condition_occurrence during day -365 through -1 days relative to index: Abnormal finding on evaluation procedure | -0.015139684 |
| 439392210 | condition_era group during day -365 through -1 days relative to index: Primary malignant neoplasm | -0.015993121 |
| 37117806502 | procedure_occurrence during day -365 through -1 days relative to index: MRI of bilateral breasts with contrast | -0.016436979 |
| 3.0034E+12 | measurement value during day -365 through -1 days relative to index: Base excess in Arterial blood by calculation (millimole per liter) | -0.017195101 |
| 192359210 | condition_era group during day -365 through -1 days relative to index: Renal failure syndrome | -0.018382394 |
| 43008998 | drug_era only per oral during day -365 through -1 days relative to index: efonidipine | -0.019230257 |
| 4316083102 | condition_occurrence during day -365 through -1 days relative to index: Skin lesion | -0.020532472 |
| 987245 | drug_era only per oral during day -365 through -1 days relative to index: lactulose | -0.022883272 |
| 744740 | drug_era only per oral during day -365 through -1 days relative to index: zolpidem | -0.023929163 |
| 4235749502 | procedure_occurrence during day -365 through -1 days relative to index: Laparoscopic-assisted anterior resection of rectum | -0.02443624 |
| 77079210 | condition_era group during day -365 through -1 days relative to index: Spinal stenosis | -0.025477383 |
| 36713032502 | procedure_occurrence during day -365 through -1 days relative to index: CT angiography of neck and chest and abdomen | -0.025643961 |
| 4201717210 | condition_era group during day -365 through -1 days relative to index: Ileostomy present | -0.027254663 |
| 321588210 | condition_era group during day -365 through -1 days relative to index: Heart disease | -0.027662618 |
| 4.18829E+12 | measurement value during day -365 through -1 days relative to index: Instrumental activity of daily living (score) | -0.028036964 |
| 961047 | drug_era only per oral during day -365 through -1 days relative to index: ranitidine | -0.029385006 |
| 80502102 | condition_occurrence during day -365 through -1 days relative to index: Osteoporosis | -0.029692931 |
| 436635210 | condition_era group during day -365 through -1 days relative to index: Primary malignant neoplasm of sigmoid colon | -0.03032727 |
| 4220313210 | condition_era group during day -365 through -1 days relative to index: Primary degenerative dementia of the Alzheimer type, senile onset | -0.03105668 |
| 40482228210 | condition_era group during day -365 through -1 days relative to index: Imaging of thorax abnormal | -0.031088422 |
| 433595210 | condition_era group during day -365 through -1 days relative to index: Edema | -0.031622937 |
| 4111018210 | condition_era group during day -365 through -1 days relative to index: Neoplasm of thorax | -0.033719401 |
| 444108210 | condition_era group during day -365 through -1 days relative to index: Finding related to sleep | -0.036197832 |
| 939976 | drug_era only per oral during day -365 through -1 days relative to index: sodium sulfate | -0.036694994 |
| 4042074210 | condition_era group during day -365 through -1 days relative to index: Blood drug level high | -0.037374709 |
| 443432210 | condition_era group during day -365 through -1 days relative to index: Impaired cognition | -0.039616509 |
| 4171549210 | condition_era group during day -365 through -1 days relative to index: Nodular goiter | -0.04014337 |
| 4043671210 | condition_era group during day -365 through -1 days relative to index: Inflammatory disorder of upper respiratory tract | -0.041882092 |
| 4289001102 | condition_occurrence during day -365 through -1 days relative to index: Acute gastroenteritis | -0.042067839 |
| 4055351210 | condition_era group during day -365 through -1 days relative to index: Polyp of gallbladder | -0.042379223 |
| 3.02581E+12 | measurement value during day -365 through -1 days relative to index: Q-T interval (Unknown unit) | -0.043574379 |
| 37310791502 | procedure_occurrence during day -365 through -1 days relative to index: CT of kidney, ureter and bladder with contrast | -0.043737534 |
| 30346210 | condition_era group during day -365 through -1 days relative to index: Primary malignant neoplasm of neck | -0.045606298 |
| 3.00628E+12 | measurement value during day -365 through -1 days relative to index: QRS axis (Unknown unit) | -0.046217549 |
| 4154801210 | condition_era group during day -365 through -1 days relative to index: Observation of sensation | -0.047369829 |
| 3.01626E+12 | measurement value during day -365 through -1 days relative to index: Waist Circumference at umbilicus by Tape measure (centimeter) | -0.048652651 |
| 443454210 | condition_era group during day -365 through -1 days relative to index: Cerebral infarction | -0.051628199 |
| 4119777210 | condition_era group during day -365 through -1 days relative to index: Ectactic vein | -0.0520669 |
| 3.01034E+12 | measurement value during day -365 through -1 days relative to index: Triiodothyronine (T3) [Mass/volume] in Serum or Plasma (nanogram per deciliter) | -0.052716601 |
| 4083519802 | observation during day -365 through -1 days relative to index: Family history of disorder | -0.056756232 |
| 4189293210 | condition_era group during day -365 through -1 days relative to index: Vascular disorder of lower extremity | -0.057270327 |
| 443381102 | condition_occurrence during day -365 through -1 days relative to index: Malignant tumor of sigmoid colon | -0.059216104 |
| 439418102 | condition_occurrence during day -365 through -1 days relative to index: Indigestion | -0.060354465 |
| 4182572802 | observation during day -365 through -1 days relative to index: Radiotherapy to abdomen | -0.06115044 |
| 1195334 | drug_era only per oral during day -365 through -1 days relative to index: choline | -0.063713866 |
| 40480615210 | condition_era group during day -365 through -1 days relative to index: Cognitive disorder | -0.068061968 |
| 195856210 | condition_era group during day -365 through -1 days relative to index: Cholangitis | -0.069628424 |
| 1545958 | drug_era only per oral during day -365 through -1 days relative to index: atorvastatin | -0.069718743 |
| 4048875210 | condition_era group during day -365 through -1 days relative to index: Senile dementia | -0.069758025 |
| 196456102 | condition_occurrence during day -365 through -1 days relative to index: Gallstone | -0.0728986 |
| 19030751 | drug_era only per oral during day -365 through -1 days relative to index: alverine | -0.073395121 |
| 40484533210 | condition_era group during day -365 through -1 days relative to index: Measurement finding below reference range | -0.073925166 |
| 3.03802E+12 | measurement value during day -365 through -1 days relative to index: Fluid balance 24 hour (Unknown unit) | -0.074216001 |
| 442793210 | condition_era group during day -365 through -1 days relative to index: Complication due to diabetes mellitus | -0.074494776 |
| 4217238502 | procedure_occurrence during day -365 through -1 days relative to index: Radioisotope scan of bone | -0.075972796 |
| 40484156210 | condition_era group during day -365 through -1 days relative to index: Malignant adenomatous neoplasm | -0.077841396 |
| 197804210 | condition_era group during day -365 through -1 days relative to index: Primary malignant neoplasm of intra-abdominal organs | -0.078169548 |
| 4154162210 | condition_era group during day -365 through -1 days relative to index: Head and neck injury | -0.080878356 |
| 4083230502 | procedure_occurrence during day -365 through -1 days relative to index: MRI of abdomen | -0.081665666 |
| 8532001 | gender = FEMALE | -0.083205952 |
| 4256761210 | condition_era group during day -365 through -1 days relative to index: Imaging result abnormal | -0.083420723 |
| 4051104802 | observation during day -365 through -1 days relative to index: No family history of | -0.084756578 |
| 4134294210 | condition_era group during day -365 through -1 days relative to index: Acute inflammatory disease | -0.084962255 |
| 4091134502 | procedure_occurrence during day -365 through -1 days relative to index: Pure tone audiometry | -0.085328768 |
| 35622827502 | procedure_occurrence during day -365 through -1 days relative to index: Radionuclide imaging of liver and/or biliary tract using radioactive isotope | -0.086587184 |
| 3.0199E+12 | measurement value during day -365 through -1 days relative to index: Erythrocyte distribution width [Ratio] by Automated count (percent) | -0.089697623 |
| 192956210 | condition_era group during day -365 through -1 days relative to index: Cholecystitis | -0.091769162 |
| 443530102 | condition_occurrence during day -365 through -1 days relative to index: Hematochezia | -0.094296938 |
| 198464210 | condition_era group during day -365 through -1 days relative to index: Incisional hernia | -0.094545313 |
| 436635102 | condition_occurrence during day -365 through -1 days relative to index: Primary malignant neoplasm of sigmoid colon | -0.097453574 |
| 4130997210 | condition_era group during day -365 through -1 days relative to index: Neoplasm of ascending colon | -0.098047137 |
| 588017 | drug_era only per oral during day -365 through -1 days relative to index: Amino Acids | -0.099862471 |
| 4310996102 | condition_occurrence during day -365 through -1 days relative to index: Ischemic stroke | -0.103226634 |
| 4087642210 | condition_era group during day -365 through -1 days relative to index: Distention of vein | -0.103614972 |
| 1334456 | drug_era only per oral during day -365 through -1 days relative to index: ramipril | -0.105840718 |
| 45765544502 | procedure_occurrence during day -365 through -1 days relative to index: CT of thyroid with contrast | -0.107694654 |
| 4.18672E+12 | measurement value during day -365 through -1 days relative to index: Basic activity of daily living (score) | -0.108386941 |
| 19136184 | drug_era only per oral during day -365 through -1 days relative to index: streptodornase | -0.109358536 |
| 4054503210 | condition_era group during day -365 through -1 days relative to index: Neoplasm of intra-abdominal organs | -0.111743774 |
| 4217838210 | condition_era group during day -365 through -1 days relative to index: Submucosal tumor of gastrointestinal tract | -0.113535689 |
| 321318102 | condition_occurrence during day -365 through -1 days relative to index: Angina pectoris | -0.113843676 |
| 133424210 | condition_era group during day -365 through -1 days relative to index: Primary malignant neoplasm of thyroid gland | -0.118379917 |
| 1332418 | drug_era only per oral during day -365 through -1 days relative to index: amlodipine | -0.121119114 |
| 4079750210 | condition_era group during day -365 through -1 days relative to index: Osteoarthritis of knee | -0.121436953 |
| 4131614210 | condition_era group during day -365 through -1 days relative to index: Neoplasm of small intestine | -0.124052985 |
| 4144684802 | observation during day -365 through -1 days relative to index: Patient referral | -0.128263499 |
| 77079102 | condition_occurrence during day -365 through -1 days relative to index: Spinal stenosis | -0.130433148 |
| 19029393 | drug_era only per oral during day -365 through -1 days relative to index: aceclofenac | -0.131657833 |
| 3.00096E+12 | measurement value during day -365 through -1 days relative to index: Hemoglobin [Mass/volume] in Blood (gram per deciliter) | -0.132673276 |
| 4244986502 | procedure_occurrence during day -365 through -1 days relative to index: CT of brain without contrast | -0.13515541 |
| 4036803502 | procedure_occurrence during day -365 through -1 days relative to index: General examination of patient | -0.138432943 |
| 1149196 | drug_era only per oral during day -365 through -1 days relative to index: cetirizine | -0.161753376 |
| 1551860 | drug_era only per oral during day -365 through -1 days relative to index: pravastatin | -0.162325702 |
| 19037833 | drug_era only per oral during day -365 through -1 days relative to index: domperidone | -0.163545419 |
| 911735 | drug_era only per oral during day -365 through -1 days relative to index: rabeprazole | -0.169181973 |
| 198809102 | condition_occurrence during day -365 through -1 days relative to index: Acute cholecystitis | -0.197970029 |
| 43009021 | drug_era only per oral during day -365 through -1 days relative to index: iron acetyl transferrin | -0.198181926 |
| 201340210 | condition_era group during day -365 through -1 days relative to index: Gastritis | -0.205394635 |
| 40481517210 | condition_era group during day -365 through -1 days relative to index: Mass of soft tissue | -0.209549363 |
| 193782210 | condition_era group during day -365 through -1 days relative to index: End-stage renal disease | -0.220017419 |
| 196931210 | condition_era group during day -365 through -1 days relative to index: Neoplasm of digestive tract | -0.227004596 |
| 4162253210 | condition_era group during day -365 through -1 days relative to index: Primary malignant neoplasm of breast | -0.234720117 |
| 4185207210 | condition_era group during day -365 through -1 days relative to index: Lesion of neck | -0.2367025 |
| 312349210 | condition_era group during day -365 through -1 days relative to index: Venous varices | -0.237351662 |
| 442793102 | condition_occurrence during day -365 through -1 days relative to index: Complication due to diabetes mellitus | -0.251342145 |
| 40479817802 | observation during day -365 through -1 days relative to index: Hospital falls risk assessment score for the elderly | -0.25799978 |
| 19095164 | drug_era only per oral during day -365 through -1 days relative to index: cholecalciferol | -0.260706928 |
| 4151121502 | procedure_occurrence during day -365 through -1 days relative to index: Low anterior resection of rectum | -0.264396111 |
| 443454102 | condition_occurrence during day -365 through -1 days relative to index: Cerebral infarction | -0.265889534 |
| 3.02008E+12 | measurement value during day -365 through -1 days relative to index: FVC pre bronchodilation measured/predicted (percent) | -0.279650523 |
| 4159963210 | condition_era group during day -365 through -1 days relative to index: Inflammatory disorder of genitourinary system | -0.29023582 |
| 2E+15 | measurement value during day -365 through -1 days relative to index: EPI-CKD eGFR, Cr-based (Unknown unit) | -0.347698312 |
| 4201717102 | condition_occurrence during day -365 through -1 days relative to index: Ileostomy present | -0.36638363 |
| 46271022102 | condition_occurrence during day -365 through -1 days relative to index: Chronic kidney disease | -0.383058201 |
| 4196958502 | procedure_occurrence during day -365 through -1 days relative to index: Laparoscopic procedure | -0.422310276 |
| 934075 | drug_era only per oral during day -365 through -1 days relative to index: azelastine | -0.491629641 |
| 4288544210 | condition_era group during day -365 through -1 days relative to index: Inguinal hernia | -0.585174487 |
| 3.00779E+12 | measurement value during day -365 through -1 days relative to index: P-R Interval (Unknown unit) | -0.762149865 |
| 3.03366E+12 | measurement value during day -365 through -1 days relative to index: Prothrombin time (PT) actual/Normal (percent) | -0.785885418 |
| 3.02456E+12 | measurement value during day -365 through -1 days relative to index: Albumin [Mass/volume] in Serum or Plasma (gram per deciliter) | -1.892293808 |
