# Supplementary material for: Development and Validation of a Prognostic Classification Model Predicting Postoperative Adverse Outcomes in Older Surgical Patients Using a Machine Learning Algorithm: Retrospective Observational Network Study
Source: J Med Internet Res. 2023 Nov 13;25:e42259. doi: 10.2196/42259 (PMC10682929; doi:10.2196/42259)
Supplement: Multimedia Appendix 8 [file jmir_v25i1e42259_app8.docx]

Selected 141 variables and covariate values of the Lasso Logistic Regression model for postoperative delirium during hospitalization

| covariateId | covariateName | covariateValue |
| --- | --- | --- |
| 1002 | age in years | 3.961905406 |
| 4181343102 | condition_occurrence during day -365 through -1 days relative to index: Malignant tumor of esophagus | 0.621157827 |
| 3.00048E+12 | measurement value during day -365 through -1 days relative to index: Glucose [Mass/volume] in Blood (milligram per deciliter) | 0.608553644 |
| 4176946210 | condition_era group during day -365 through -1 days relative to index: Inflammatory disorder of musculoskeletal system | 0.579612923 |
| 3.02707E+12 | measurement value during day -365 through -1 days relative to index: Posterior tibial artery - right Systolic blood pressure (millimeter mercury column) | 0.569011882 |
| 725131 | drug_era only per oral during day -365 through -1 days relative to index: mirtazapine | 0.554373871 |
| 3.01261E+12 | measurement value during day -365 through -1 days relative to index: Segmented neutrophils/100 leukocytes in Blood by Automated count (percent) | 0.544418346 |
| 761012210 | condition_era group during day -365 through -1 days relative to index: Lesion of vertebra | 0.480120949 |
| 4103495210 | condition_era group during day -365 through -1 days relative to index: Aneurysm of artery of trunk | 0.476451341 |
| 36713032502 | procedure_occurrence during day -365 through -1 days relative to index: CT angiography of neck and chest and abdomen | 0.344736259 |
| 77079210 | condition_era group during day -365 through -1 days relative to index: Spinal stenosis | 0.326458854 |
| 77670102 | condition_occurrence during day -365 through -1 days relative to index: Chest pain | 0.297557525 |
| 3.03617E+12 | measurement value during day -365 through -1 days relative to index: Chloride [Moles/volume] in Cerebral spinal fluid (millimole per liter) | 0.296095832 |
| 3.02046E+12 | measurement value during day -365 through -1 days relative to index: C reactive protein [Mass/volume] in Serum or Plasma (milligram per deciliter) | 0.261463073 |
| 4154630210 | condition_era group during day -365 through -1 days relative to index: Malignant neoplasm of genitourinary organ | 0.253942612 |
| 3.02626E+12 | measurement value during day -365 through -1 days relative to index: Q-T interval corrected (Unknown unit) | 0.229898095 |
| 3.0165E+12 | measurement value during day -365 through -1 days relative to index: Oxygen saturation in Arterial blood (percent) | 0.219454687 |
| 1114220 | drug_era only per oral during day -365 through -1 days relative to index: naloxone | 0.210246643 |
| 443454102 | condition_occurrence during day -365 through -1 days relative to index: Cerebral infarction | 0.207837781 |
| 4161393502 | procedure_occurrence during day -365 through -1 days relative to index: MRI of pelvis with contrast | 0.203273724 |
| 194589210 | condition_era group during day -365 through -1 days relative to index: Primary malignant neoplasm of biliary tract | 0.201076311 |
| 4177244210 | condition_era group during day -365 through -1 days relative to index: Malignant tumor of female genital organ | 0.193676825 |
| 45765716502 | procedure_occurrence during day -365 through -1 days relative to index: MRI of head and neck with contrast | 0.190525364 |
| 4180790102 | condition_occurrence during day -365 through -1 days relative to index: Malignant tumor of colon | 0.18828527 |
| 443784210 | condition_era group during day -365 through -1 days relative to index: Vascular disorder | 0.186631198 |
| 4299435802 | observation during day -365 through -1 days relative to index: Site of distant metastasis | 0.181972551 |
| 2E+15 | measurement value during day -365 through -1 days relative to index: Indicator of Surgery Cancer (score) | 0.169475141 |
| 745466 | drug_era only per oral during day -365 through -1 days relative to index: valproate | 0.16848543 |
| 1103314 | drug_era only per oral during day -365 through -1 days relative to index: tramadol | 0.165851536 |
| 4335825502 | procedure_occurrence during day -365 through -1 days relative to index: Transthoracic echocardiography | 0.157985574 |
| 4306317502 | procedure_occurrence during day -365 through -1 days relative to index: CT angiography of coronary arteries | 0.151715564 |
| 36713053502 | procedure_occurrence during day -365 through -1 days relative to index: MR angiography of brain and neck without contrast | 0.135868652 |
| 791967 | drug_era only per oral during day -365 through -1 days relative to index: lorazepam | 0.127925014 |
| 439383210 | condition_era group during day -365 through -1 days relative to index: Vertigo | 0.121858994 |
| 1124957 | drug_era only per oral during day -365 through -1 days relative to index: oxycodone | 0.119810433 |
| 4022173502 | procedure_occurrence during day -365 through -1 days relative to index: Transfusion of red blood cells | 0.112030105 |
| 4329041102 | condition_occurrence during day -365 through -1 days relative to index: Pain | 0.109399087 |
| 4143065102 | condition_occurrence during day -365 through -1 days relative to index: Involuntary movement | 0.108010648 |
| 373747210 | condition_era group during day -365 through -1 days relative to index: Extrapyramidal disease | 0.105298784 |
| 26638102 | condition_occurrence during day -365 through -1 days relative to index: Primary malignant neoplasm of esophagus | 0.101244839 |
| 4329041210 | condition_era group during day -365 through -1 days relative to index: Pain | 0.100979259 |
| 4058335502 | procedure_occurrence during day -365 through -1 days relative to index: CT of chest | 0.095609754 |
| 77079102 | condition_occurrence during day -365 through -1 days relative to index: Spinal stenosis | 0.091128469 |
| 974166 | drug_era only per oral during day -365 through -1 days relative to index: hydrochlorothiazide | 0.089533384 |
| 317585102 | condition_occurrence during day -365 through -1 days relative to index: Aortic aneurysm | 0.087802666 |
| 4239975210 | condition_era group during day -365 through -1 days relative to index: Myocardial disease | 0.083457673 |
| 4178904210 | condition_era group during day -365 through -1 days relative to index: Body temperature above reference range | 0.079916043 |
| 3.0083E+12 | measurement value during day -365 through -1 days relative to index: Osmolality of Serum or Plasma (milliosmole per kilogram) | 0.076640806 |
| 37109061502 | procedure_occurrence during day -365 through -1 days relative to index: CT angiography of brain and neck artery | 0.075827312 |
| 4129891210 | condition_era group during day -365 through -1 days relative to index: Neoplasm of retroperitoneum | 0.075633873 |
| 317576102 | condition_occurrence during day -365 through -1 days relative to index: Coronary arteriosclerosis | 0.074449451 |
| 4202832502 | procedure_occurrence during day -365 through -1 days relative to index: Intubation | 0.074223504 |
| 313217102 | condition_occurrence during day -365 through -1 days relative to index: Atrial fibrillation | 0.0711327 |
| 4103523502 | procedure_occurrence during day -365 through -1 days relative to index: Laryngoscopy | 0.069899246 |
| 1326303 | drug_era only per oral during day -365 through -1 days relative to index: digoxin | 0.06768323 |
| 37117305502 | procedure_occurrence during day -365 through -1 days relative to index: CT of abdomen and pelvis without contrast | 0.061943283 |
| 317009102 | condition_occurrence during day -365 through -1 days relative to index: Asthma | 0.061104525 |
| 4068261210 | condition_era group during day -365 through -1 days relative to index: Bile duct proliferation | 0.058023891 |
| 1332418 | drug_era only per oral during day -365 through -1 days relative to index: amlodipine | 0.047323203 |
| 372604210 | condition_era group during day -365 through -1 days relative to index: Movement disorder | 0.045705317 |
| 993631 | drug_era only per oral during day -365 through -1 days relative to index: magnesium oxide | 0.041879482 |
| 4028253210 | condition_era group during day -365 through -1 days relative to index: Neoplasm of digestive system | 0.039827741 |
| 4304092502 | procedure_occurrence during day -365 through -1 days relative to index: CT of abdomen and pelvis | 0.038832207 |
| 1139042 | drug_era only per oral during day -365 through -1 days relative to index: acetylcysteine | 0.038788264 |
| 4024561210 | condition_era group during day -365 through -1 days relative to index: Pain in lower limb | 0.034955514 |
| 26638210 | condition_era group during day -365 through -1 days relative to index: Primary malignant neoplasm of esophagus | 0.032698674 |
| 3.00567E+12 | measurement value during day -365 through -1 days relative to index: Hemoglobin A1c/Hemoglobin.total in Blood by HPLC (percent) | 0.028365284 |
| 4.0482E+13 | measurement value during day -365 through -1 days relative to index: Geriatric depression scale short form (score) | 0.026773953 |
| 45765485502 | procedure_occurrence during day -365 through -1 days relative to index: CT angiography of neck vessels | 0.025074121 |
| 1195334 | drug_era only per oral during day -365 through -1 days relative to index: choline | 0.024586048 |
| 4329640502 | procedure_occurrence during day -365 through -1 days relative to index: MRI of liver with contrast | 0.022486734 |
| 1361711 | drug_era only per oral during day -365 through -1 days relative to index: nitroglycerin | 0.02005411 |
| 437663210 | condition_era group during day -365 through -1 days relative to index: Fever | 0.014338255 |
| 744740 | drug_era only per oral during day -365 through -1 days relative to index: zolpidem | 0.010131211 |
| 136788102 | condition_occurrence during day -365 through -1 days relative to index: Spinal stenosis of lumbar region | 0.007836077 |
| 4200516210 | condition_era group during day -365 through -1 days relative to index: Lesion of brain | 0.004675807 |
| 1125315 | drug_era only per oral during day -365 through -1 days relative to index: acetaminophen | 0.002545415 |
| 437663102 | condition_occurrence during day -365 through -1 days relative to index: Fever | 0.001832092 |
| 4047791210 | condition_era group during day -365 through -1 days relative to index: Abnormal body temperature | 0.001312203 |
| 4088035802 | observation during day -365 through -1 days relative to index: Screening status | -0.00771114 |
| 46271022102 | condition_occurrence during day -365 through -1 days relative to index: Chronic kidney disease | -0.009202402 |
| 4041664210 | condition_era group during day -365 through -1 days relative to index: Difficulty breathing | -0.014715546 |
| 939976 | drug_era only per oral during day -365 through -1 days relative to index: sodium sulfate | -0.020157987 |
| 924566 | drug_era only per oral during day -365 through -1 days relative to index: tamsulosin | -0.027801278 |
| 192438210 | condition_era group during day -365 through -1 days relative to index: Abdominal mass | -0.031136733 |
| 4051104802 | observation during day -365 through -1 days relative to index: No family history of | -0.032170417 |
| 40479817802 | observation during day -365 through -1 days relative to index: Hospital falls risk assessment score for the elderly | -0.034817589 |
| 4145825210 | condition_era group during day -365 through -1 days relative to index: Anorectal disorder | -0.036724076 |
| 40481841210 | condition_era group during day -365 through -1 days relative to index: Measurement finding outside reference range | -0.037890701 |
| 381591210 | condition_era group during day -365 through -1 days relative to index: Cerebrovascular disease | -0.039561989 |
| 966991 | drug_era only per oral during day -365 through -1 days relative to index: simethicone | -0.039992463 |
| 40481925802 | observation during day -365 through -1 days relative to index: No history of clinical finding in subject | -0.042778555 |
| 4181063210 | condition_era group during day -365 through -1 days relative to index: Inflammation of specific body organs | -0.046121927 |
| 1367500 | drug_era only per oral during day -365 through -1 days relative to index: losartan | -0.055689068 |
| 255848102 | condition_occurrence during day -365 through -1 days relative to index: Pneumonia | -0.061987601 |
| 45765544502 | procedure_occurrence during day -365 through -1 days relative to index: CT of thyroid with contrast | -0.064780622 |
| 443387102 | condition_occurrence during day -365 through -1 days relative to index: Malignant tumor of stomach | -0.065006523 |
| 198809210 | condition_era group during day -365 through -1 days relative to index: Acute cholecystitis | -0.082788919 |
| 432545210 | condition_era group during day -365 through -1 days relative to index: Bacterial infectious disease | -0.083398279 |
| 197500210 | condition_era group during day -365 through -1 days relative to index: Primary malignant neoplasm of colon | -0.087565697 |
| 135772210 | condition_era group during day -365 through -1 days relative to index: Goiter | -0.091790528 |
| 4115576210 | condition_era group during day -365 through -1 days relative to index: Lesion of gallbladder | -0.091972675 |
| 197804210 | condition_era group during day -365 through -1 days relative to index: Primary malignant neoplasm of intra-abdominal organs | -0.093119246 |
| 4111018210 | condition_era group during day -365 through -1 days relative to index: Neoplasm of thorax | -0.096574875 |
| 1797513 | drug_era only per oral during day -365 through -1 days relative to index: ciprofloxacin | -0.101996271 |
| 36713047502 | procedure_occurrence during day -365 through -1 days relative to index: Low dose computed tomography of chest without contrast | -0.111233273 |
| 40479625210 | condition_era group during day -365 through -1 days relative to index: Atherosclerosis of artery | -0.121752955 |
| 3.03628E+12 | measurement value during day -365 through -1 days relative to index: Body height (centimeter) | -0.13840445 |
| 19088167 | drug_era only per oral during day -365 through -1 days relative to index: ambroxol | -0.146955375 |
| 4145627210 | condition_era group during day -365 through -1 days relative to index: Biliary calculus | -0.151259516 |
| 73553210 | condition_era group during day -365 through -1 days relative to index: Arthropathy | -0.155076375 |
| 766814 | drug_era only per oral during day -365 through -1 days relative to index: quetiapine | -0.162157058 |
| 321318210 | condition_era group during day -365 through -1 days relative to index: Angina pectoris | -0.167196444 |
| 4134595210 | condition_era group during day -365 through -1 days relative to index: Chronic disease of genitourinary system | -0.167661473 |
| 2E+15 | measurement value during day -365 through -1 days relative to index: EPI-CKD eGFR, Cr-based (Unknown unit) | -0.169822985 |
| 3.01563E+12 | measurement value during day -365 through -1 days relative to index: Carbon dioxide, total [Moles/volume] in Serum or Plasma (millimole per liter) | -0.170459272 |
| 3.00707E+12 | measurement value during day -365 through -1 days relative to index: Cholesterol in HDL [Mass/volume] in Serum or Plasma (milligram per deciliter) | -0.170784114 |
| 4190185210 | condition_era group during day -365 through -1 days relative to index: Inflammatory disorder of digestive system | -0.179832253 |
| 929887 | drug_era only per oral during day -365 through -1 days relative to index: lansoprazole | -0.182276563 |
| 197032210 | condition_era group during day -365 through -1 days relative to index: Hyperplasia of prostate | -0.212391703 |
| 192956210 | condition_era group during day -365 through -1 days relative to index: Cholecystitis | -0.218607193 |
| 4113547210 | condition_era group during day -365 through -1 days relative to index: Lesion of stomach | -0.222149739 |
| 3.01432E+12 | measurement value during day -365 through -1 days relative to index: Urine output (Non-specific) | -0.233870793 |
| 939506 | drug_era only per oral during day -365 through -1 days relative to index: sodium bicarbonate | -0.323788179 |
| 443387210 | condition_era group during day -365 through -1 days relative to index: Malignant tumor of stomach | -0.342973265 |
| 435506210 | condition_era group during day -365 through -1 days relative to index: Benign neoplastic disease | -0.363386691 |
| 197500102 | condition_occurrence during day -365 through -1 days relative to index: Primary malignant neoplasm of colon | -0.378166145 |
| 1337620 | drug_era only per oral during day -365 through -1 days relative to index: capecitabine | -0.379139501 |
| 443530102 | condition_occurrence during day -365 through -1 days relative to index: Hematochezia | -0.388550308 |
| 3.04311E+12 | measurement value during day -365 through -1 days relative to index: Platelet mean volume [Entitic volume] in Blood by Automated count (femtoliter) | -0.474991196 |
| 192359210 | condition_era group during day -365 through -1 days relative to index: Renal failure syndrome | -0.492669877 |
| 4267297210 | condition_era group during day -365 through -1 days relative to index: Acute cholangitis | -0.516417084 |
| 8532001 | gender = FEMALE | -0.547066579 |
| 2E+15 | measurement value during day -365 through -1 days relative to index: PEF pre bronchodilation measured/predicted (percent) | -0.649964615 |
| 4148972502 | procedure_occurrence during day -365 through -1 days relative to index: Extubation of trachea | -0.700475187 |
| 81251210 | condition_era group during day -365 through -1 days relative to index: Neoplasm of breast | -0.71642237 |
| 3.03366E+12 | measurement value during day -365 through -1 days relative to index: Prothrombin time (PT) actual/Normal (percent) | -0.779283577 |
| 196456210 | condition_era group during day -365 through -1 days relative to index: Gallstone | -0.812045387 |
| 3.03751E+12 | measurement value during day -365 through -1 days relative to index: Lymphocytes/100 leukocytes in Blood by Automated count (percent) | -1.082371077 |
| 4288544210 | condition_era group during day -365 through -1 days relative to index: Inguinal hernia | -1.085465483 |
| 3.02456E+12 | measurement value during day -365 through -1 days relative to index: Albumin [Mass/volume] in Serum or Plasma (gram per deciliter) | -1.313585515 |
