# Supplementary material for: Development and Validation of a Prognostic Classification Model Predicting Postoperative Adverse Outcomes in Older Surgical Patients Using a Machine Learning Algorithm: Retrospective Observational Network Study
Source: J Med Internet Res. 2023 Nov 13;25:e42259. doi: 10.2196/42259 (PMC10682929; doi:10.2196/42259)
Supplement: Multimedia Appendix 9 [file jmir_v25i1e42259_app9.docx]

Selected 465 variables and covariate values of the Lasso Logistic Regression model for prolonged postoperative stay

| covariateId | covariateName | covariateValue |
| --- | --- | --- |
| 4181343102 | condition_occurrence during day -365 through -1 days relative to index: Malignant tumor of esophagus | 0.948223791 |
| 4155446502 | procedure_occurrence during day -365 through -1 days relative to index: Plain film of head | 0.86967374 |
| 26638102 | condition_occurrence during day -365 through -1 days relative to index: Primary malignant neoplasm of esophagus | 0.861375091 |
| 3.02702E+12 | measurement value during day -365 through -1 days relative to index: Heart rate (Non-specific) | 0.823941166 |
| 3.01261E+12 | measurement value during day -365 through -1 days relative to index: Segmented neutrophils/100 leukocytes in Blood by Automated count (percent) | 0.802434372 |
| 36713363102 | condition_occurrence during day -365 through -1 days relative to index: Primary adenocarcinoma of head of pancreas | 0.752648516 |
| 3.02046E+12 | measurement value during day -365 through -1 days relative to index: C reactive protein [Mass/volume] in Serum or Plasma (milligram per deciliter) | 0.744973203 |
| 193242210 | condition_era group during day -365 through -1 days relative to index: Perforation of intestine | 0.676752742 |
| 3.03477E+12 | measurement value during day -365 through -1 days relative to index: Posterior tibial artery - right Diastolic blood pressure (millimeter mercury column) | 0.659874717 |
| 3.0199E+12 | measurement value during day -365 through -1 days relative to index: Erythrocyte distribution width [Ratio] by Automated count (percent) | 0.55825351 |
| 4103495210 | condition_era group during day -365 through -1 days relative to index: Aneurysm of artery of trunk | 0.554771128 |
| 1796458 | drug_era only per oral during day -365 through -1 days relative to index: cefdinir | 0.554121498 |
| 4068261210 | condition_era group during day -365 through -1 days relative to index: Bile duct proliferation | 0.541003273 |
| 3.02707E+12 | measurement value during day -365 through -1 days relative to index: Posterior tibial artery - right Systolic blood pressure (millimeter mercury column) | 0.53347274 |
| 3.02473E+12 | measurement value during day -365 through -1 days relative to index: MCV [Entitic volume] (femtoliter) | 0.526188682 |
| 19042778 | drug_era only per oral during day -365 through -1 days relative to index: triflusal | 0.476110516 |
| 4143249802 | observation during day -365 through -1 days relative to index: Determination of acuity level | 0.475061006 |
| 4239794102 | condition_occurrence during day -365 through -1 days relative to index: Calculus of hepatic duct | 0.462997221 |
| 4163971502 | procedure_occurrence during day -365 through -1 days relative to index: Laparoscopic cholecystectomy | 0.456279557 |
| 3.00091E+12 | measurement value during day -365 through -1 days relative to index: Leukocytes [#/volume] in Blood by Automated count (thousand per microliter) | 0.437741126 |
| 36713285502 | procedure_occurrence during day -365 through -1 days relative to index: CT of lumbar spine without contrast | 0.421445755 |
| 4111798210 | condition_era group during day -365 through -1 days relative to index: Neoplasm of digestive organ | 0.416333022 |
| 4299435802 | observation during day -365 through -1 days relative to index: Site of distant metastasis | 0.415139365 |
| 924939 | drug_era only per oral during day -365 through -1 days relative to index: bisacodyl | 0.412936094 |
| 317585102 | condition_occurrence during day -365 through -1 days relative to index: Aortic aneurysm | 0.408813034 |
| 4130829502 | procedure_occurrence during day -365 through -1 days relative to index: Platelet transfusion | 0.385203953 |
| 761012210 | condition_era group during day -365 through -1 days relative to index: Lesion of vertebra | 0.363561239 |
| 444035210 | condition_era group during day -365 through -1 days relative to index: Incontinence | 0.360144086 |
| 433811210 | condition_era group during day -365 through -1 days relative to index: Hydronephrosis | 0.342132872 |
| 4.07738E+13 | measurement value during day -365 through -1 days relative to index: Erythrocyte Distribution Width \| Red Blood Cells (percent) | 0.32106251 |
| 373718210 | condition_era group during day -365 through -1 days relative to index: Neoplasm of connective tissues | 0.319163827 |
| 4129885210 | condition_era group during day -365 through -1 days relative to index: Neoplasm of common bile duct | 0.311580427 |
| 4161393502 | procedure_occurrence during day -365 through -1 days relative to index: MRI of pelvis with contrast | 0.303520151 |
| 3.02413E+12 | measurement value during day -365 through -1 days relative to index: Bilirubin.total [Mass/volume] in Serum or Plasma (milligram per deciliter) | 0.282932618 |
| 1707164 | drug_era only per oral during day -365 through -1 days relative to index: metronidazole | 0.28233633 |
| 4223530102 | condition_occurrence during day -365 through -1 days relative to index: Spondylitis | 0.280229484 |
| 4115105210 | condition_era group during day -365 through -1 days relative to index: Mass of respiratory structure | 0.277921471 |
| 196152102 | condition_occurrence during day -365 through -1 days relative to index: Peritonitis | 0.277315359 |
| 443392210 | condition_era group during day -365 through -1 days relative to index: Malignant neoplastic disease | 0.276543123 |
| 4024561102 | condition_occurrence during day -365 through -1 days relative to index: Pain in lower limb | 0.273991073 |
| 4287399210 | condition_era group during day -365 through -1 days relative to index: Retroperitoneal mass | 0.272954522 |
| 435613210 | condition_era group during day -365 through -1 days relative to index: Cellulitis | 0.272244118 |
| 31317210 | condition_era group during day -365 through -1 days relative to index: Dysphagia | 0.271560726 |
| 1114220 | drug_era only per oral during day -365 through -1 days relative to index: naloxone | 0.270752009 |
| 197304210 | condition_era group during day -365 through -1 days relative to index: Ulcer of lower extremity | 0.267358509 |
| 4213310210 | condition_era group during day -365 through -1 days relative to index: Degenerative disease of the central nervous system | 0.262675531 |
| 967861 | drug_era only per oral during day -365 through -1 days relative to index: magnesium citrate | 0.259899157 |
| 36713032502 | procedure_occurrence during day -365 through -1 days relative to index: CT angiography of neck and chest and abdomen | 0.256062443 |
| 24134210 | condition_era group during day -365 through -1 days relative to index: Neck pain | 0.254816305 |
| 4329640502 | procedure_occurrence during day -365 through -1 days relative to index: MRI of liver with contrast | 0.242156347 |
| 320739210 | condition_era group during day -365 through -1 days relative to index: Dissection of aorta | 0.234613222 |
| 4161028102 | condition_occurrence during day -365 through -1 days relative to index: Adenocarcinoma of prostate | 0.234234485 |
| 4335825502 | procedure_occurrence during day -365 through -1 days relative to index: Transthoracic echocardiography | 0.231830823 |
| 3.00096E+12 | measurement value during day -365 through -1 days relative to index: Hemoglobin [Mass/volume] in Blood (gram per deciliter) | 0.228059343 |
| 135526210 | condition_era group during day -365 through -1 days relative to index: Spinal cord disease | 0.225006728 |
| 4112603210 | condition_era group during day -365 through -1 days relative to index: Tumor of esophagus, stomach and duodenum | 0.223772692 |
| 19037833 | drug_era only per oral during day -365 through -1 days relative to index: domperidone | 0.219034788 |
| 40482784210 | condition_era group during day -365 through -1 days relative to index: Malignant neoplasm of skeletal system | 0.219013032 |
| 1002 | age in years | 0.216528538 |
| 789578 | drug_era only per oral during day -365 through -1 days relative to index: levodopa | 0.215722425 |
| 1526475 | drug_era only per oral during day -365 through -1 days relative to index: ezetimibe | 0.209428542 |
| 37109138502 | procedure_occurrence during day -365 through -1 days relative to index: CT angiography of bilateral lower extremities | 0.208745357 |
| 45765716502 | procedure_occurrence during day -365 through -1 days relative to index: MRI of head and neck with contrast | 0.203872631 |
| 4198764502 | procedure_occurrence during day -365 through -1 days relative to index: CT of brain perfusion | 0.203571771 |
| 4304092502 | procedure_occurrence during day -365 through -1 days relative to index: CT of abdomen and pelvis | 0.202966545 |
| 4068155210 | condition_era group during day -365 through -1 days relative to index: Atrial arrhythmia | 0.202244475 |
| 4311439210 | condition_era group during day -365 through -1 days relative to index: Sarcoma | 0.201303015 |
| 4047650210 | condition_era group during day -365 through -1 days relative to index: Neoplasm of ovary | 0.194897949 |
| 4143867210 | condition_era group during day -365 through -1 days relative to index: Small bowel adhesions | 0.194895359 |
| 36879181 | drug_era only per oral during day -365 through -1 days relative to index: streptococcus faecalis | 0.192093094 |
| 4205256210 | condition_era group during day -365 through -1 days relative to index: Mass of lower limb | 0.190562493 |
| 436222210 | condition_era group during day -365 through -1 days relative to index: Altered mental status | 0.189745675 |
| 4129389210 | condition_era group during day -365 through -1 days relative to index: Gastrointestinal obstruction | 0.18836449 |
| 4202832502 | procedure_occurrence during day -365 through -1 days relative to index: Intubation | 0.184821119 |
| 791967 | drug_era only per oral during day -365 through -1 days relative to index: lorazepam | 0.180574427 |
| 19066992 | drug_era only per oral during day -365 through -1 days relative to index: silymarin | 0.180090025 |
| 4167096210 | condition_era group during day -365 through -1 days relative to index: Osteitis | 0.179692273 |
| 4090425210 | condition_era group during day -365 through -1 days relative to index: Altered sensation of skin | 0.176830011 |
| 3.00815E+12 | measurement value during day -365 through -1 days relative to index: Bicarbonate [Moles/volume] in Arterial blood (millimole per liter) | 0.173620622 |
| 3.01641E+12 | measurement value during day -365 through -1 days relative to index: Fibrinogen [Mass/volume] in Platelet poor plasma by Coagulation assay (milligram per deciliter) | 0.170544321 |
| 1501700 | drug_era only per oral during day -365 through -1 days relative to index: levothyroxine | 0.163039874 |
| 4178673210 | condition_era group during day -365 through -1 days relative to index: Congenital anomaly of lower trunk | 0.161992189 |
| 4107244210 | condition_era group during day -365 through -1 days relative to index: Adhesion of intestine | 0.159911794 |
| 4179720210 | condition_era group during day -365 through -1 days relative to index: Primary malignant neoplasm of gastrointestinal tract | 0.155130642 |
| 4176946210 | condition_era group during day -365 through -1 days relative to index: Inflammatory disorder of musculoskeletal system | 0.154281719 |
| 4329041102 | condition_occurrence during day -365 through -1 days relative to index: Pain | 0.153345994 |
| 924566 | drug_era only per oral during day -365 through -1 days relative to index: tamsulosin | 0.153275489 |
| 376647210 | condition_era group during day -365 through -1 days relative to index: Primary malignant neoplasm of soft tissues | 0.152498592 |
| 377091210 | condition_era group during day -365 through -1 days relative to index: Seizure | 0.150274299 |
| 321314102 | condition_occurrence during day -365 through -1 days relative to index: Abdominal aortic aneurysm without rupture | 0.148653517 |
| 3.01368E+12 | measurement value during day -365 through -1 days relative to index: Urea nitrogen [Mass/volume] in Serum or Plasma (milligram per deciliter) | 0.146644291 |
| 4083230502 | procedure_occurrence during day -365 through -1 days relative to index: MRI of abdomen | 0.141254035 |
| 4.08103E+12 | measurement value during day -365 through -1 days relative to index: Aortic pressure (millimeter mercury column) | 0.140758964 |
| 4188043102 | condition_occurrence during day -365 through -1 days relative to index: Mechanical ileus | 0.139524234 |
| 1749008 | drug_era only per oral during day -365 through -1 days relative to index: cefpodoxime | 0.137188804 |
| 81902102 | condition_occurrence during day -365 through -1 days relative to index: Urinary tract infectious disease | 0.136291594 |
| 43009072 | drug_era only per oral during day -365 through -1 days relative to index: zaltoprofen | 0.135292441 |
| 4177244210 | condition_era group during day -365 through -1 days relative to index: Malignant tumor of female genital organ | 0.134873847 |
| 4181345210 | condition_era group during day -365 through -1 days relative to index: Malignant tumor of biliary tract | 0.133644705 |
| 45757798210 | condition_era group during day -365 through -1 days relative to index: Proliferative retinopathy | 0.132604178 |
| 4316083210 | condition_era group during day -365 through -1 days relative to index: Skin lesion | 0.132482077 |
| 19009145 | drug_era only per oral during day -365 through -1 days relative to index: mequitazine | 0.131326156 |
| 40491001210 | condition_era group during day -365 through -1 days relative to index: Malignant neoplasm of digestive system | 0.131204163 |
| 4044391210 | condition_era group during day -365 through -1 days relative to index: Neuropathy due to diabetes mellitus | 0.12986296 |
| 37208245102 | condition_occurrence during day -365 through -1 days relative to index: Primary adenocarcinoma of descending colon | 0.129713474 |
| 138525210 | condition_era group during day -365 through -1 days relative to index: Pain in limb | 0.129188731 |
| 1525215 | drug_era only per oral during day -365 through -1 days relative to index: pioglitazone | 0.128340467 |
| 4035234502 | procedure_occurrence during day -365 through -1 days relative to index: Transfusion of platelet concentrate | 0.124200405 |
| 1597756 | drug_era only per oral during day -365 through -1 days relative to index: glimepiride | 0.123112015 |
| 437312210 | condition_era group during day -365 through -1 days relative to index: Bleeding | 0.122623527 |
| 19025198 | drug_era only per oral during day -365 through -1 days relative to index: pinaverium | 0.119801387 |
| 37016239102 | condition_occurrence during day -365 through -1 days relative to index: Primary adenocarcinoma of rectosigmoid junction | 0.118794702 |
| 1310149 | drug_era only per oral during day -365 through -1 days relative to index: warfarin | 0.117229694 |
| 993631 | drug_era only per oral during day -365 through -1 days relative to index: magnesium oxide | 0.116168025 |
| 3.01518E+12 | measurement value during day -365 through -1 days relative to index: Erythrocyte sedimentation rate (millimeter per hour) | 0.114061477 |
| 4095434210 | condition_era group during day -365 through -1 days relative to index: Malignant neoplasm of hepatic duct | 0.113516716 |
| 4180794210 | condition_era group during day -365 through -1 days relative to index: Malignant retroperitoneal tumor | 0.113214192 |
| 4244986502 | procedure_occurrence during day -365 through -1 days relative to index: CT of brain without contrast | 0.113128844 |
| 777221 | drug_era only per oral during day -365 through -1 days relative to index: hydroxyzine | 0.112868403 |
| 1518254 | drug_era only per oral during day -365 through -1 days relative to index: dexamethasone | 0.11192654 |
| 4311817502 | procedure_occurrence during day -365 through -1 days relative to index: Fiberoptic bronchoscopy | 0.111033649 |
| 3.00048E+12 | measurement value during day -365 through -1 days relative to index: Glucose [Mass/volume] in Blood (milligram per deciliter) | 0.108786232 |
| 36878752 | drug_era only per oral during day -365 through -1 days relative to index: smectite | 0.106194165 |
| 4215685802 | observation during day -365 through -1 days relative to index: Past history of procedure | 0.1049753 |
| 3.00181E+12 | measurement value during day -365 through -1 days relative to index: Brachial artery - right Diastolic blood pressure (millimeter mercury column) | 0.10311178 |
| 314666210 | condition_era group during day -365 through -1 days relative to index: Old myocardial infarction | 0.102996546 |
| 26638210 | condition_era group during day -365 through -1 days relative to index: Primary malignant neoplasm of esophagus | 0.099328947 |
| 3.0276E+12 | measurement value during day -365 through -1 days relative to index: Mean blood pressure (millimeter mercury column) | 0.099260132 |
| 4177236210 | condition_era group during day -365 through -1 days relative to index: Malignant tumor of pelvis | 0.097423384 |
| 19137056 | drug_era only per oral during day -365 through -1 days relative to index: theobromine | 0.09647675 |
| 4174764102 | condition_occurrence during day -365 through -1 days relative to index: Late gastric cancer | 0.096185317 |
| 4134593210 | condition_era group during day -365 through -1 days relative to index: Chronic digestive system disorder | 0.095932711 |
| 4008576102 | condition_occurrence during day -365 through -1 days relative to index: Diabetes mellitus without complication | 0.095829561 |
| 987245 | drug_era only per oral during day -365 through -1 days relative to index: lactulose | 0.0947 |
| 42596177210 | condition_era group during day -365 through -1 days relative to index: Mass of intestine | 0.093942731 |
| 43009013 | drug_era only per oral during day -365 through -1 days relative to index: beraprost sodium | 0.09366908 |
| 19100759 | drug_era only per oral during day -365 through -1 days relative to index: tiropramide | 0.093249952 |
| 4113998210 | condition_era group during day -365 through -1 days relative to index: Mass of urogenital structure | 0.092977687 |
| 4193176210 | condition_era group during day -365 through -1 days relative to index: Musculoskeletal infective disorder | 0.091739836 |
| 19029393 | drug_era only per oral during day -365 through -1 days relative to index: aceclofenac | 0.091049944 |
| 4045736210 | condition_era group during day -365 through -1 days relative to index: Posterior cerebral circulation infarction | 0.090735954 |
| 4203098210 | condition_era group during day -365 through -1 days relative to index: Mass of back | 0.090481335 |
| 1118084 | drug_era only per oral during day -365 through -1 days relative to index: celecoxib | 0.090471198 |
| 4181638502 | procedure_occurrence during day -365 through -1 days relative to index: Cardiovascular examination and evaluation | 0.088862135 |
| 4181351210 | condition_era group during day -365 through -1 days relative to index: Malignant tumor of ovary | 0.088412828 |
| 19030059 | drug_era only per oral during day -365 through -1 days relative to index: alginic acid | 0.088202866 |
| 4324448502 | procedure_occurrence during day -365 through -1 days relative to index: CT of pancreas with contrast | 0.087184497 |
| 950637 | drug_era only per oral during day -365 through -1 days relative to index: tacrolimus | 0.085800365 |
| 4143397210 | condition_era group during day -365 through -1 days relative to index: Mass of pancreas | 0.085774112 |
| 19059796 | drug_era only per oral during day -365 through -1 days relative to index: gliclazide | 0.083087762 |
| 4162038210 | condition_era group during day -365 through -1 days relative to index: Occlusion of artery | 0.077753396 |
| 1136422 | drug_era only per oral during day -365 through -1 days relative to index: levocetirizine | 0.077557606 |
| 40481517210 | condition_era group during day -365 through -1 days relative to index: Mass of soft tissue | 0.077223477 |
| 36684472210 | condition_era group during day -365 through -1 days relative to index: Primary malignant neoplasm of abdomen | 0.077184835 |
| 4137275210 | condition_era group during day -365 through -1 days relative to index: Vasculitis | 0.074192026 |
| 443784210 | condition_era group during day -365 through -1 days relative to index: Vascular disorder | 0.070896824 |
| 937439 | drug_era only per oral during day -365 through -1 days relative to index: bethanechol | 0.070603536 |
| 134736210 | condition_era group during day -365 through -1 days relative to index: Backache | 0.070027848 |
| 4058335502 | procedure_occurrence during day -365 through -1 days relative to index: CT of chest | 0.06933736 |
| 906780 | drug_era only per oral during day -365 through -1 days relative to index: metoclopramide | 0.068592593 |
| 4026023102 | condition_occurrence during day -365 through -1 days relative to index: Ileal adhesions | 0.065487411 |
| 3.03617E+12 | measurement value during day -365 through -1 days relative to index: Chloride [Moles/volume] in Cerebral spinal fluid (millimole per liter) | 0.064163533 |
| 1300978 | drug_era only per oral during day -365 through -1 days relative to index: megestrol | 0.06339164 |
| 1036228 | drug_era only per oral during day -365 through -1 days relative to index: sucralfate | 0.062661419 |
| 4027369210 | condition_era group during day -365 through -1 days relative to index: Vascular disease of abdomen | 0.060877138 |
| 4177183210 | condition_era group during day -365 through -1 days relative to index: Perforation of colon | 0.058885313 |
| 4187096210 | condition_era group during day -365 through -1 days relative to index: Fracture of lower limb | 0.057858609 |
| 312437102 | condition_occurrence during day -365 through -1 days relative to index: Dyspnea | 0.055468138 |
| 374371210 | condition_era group during day -365 through -1 days relative to index: Stenosis of precerebral artery | 0.054547373 |
| 3.00142E+12 | measurement value during day -365 through -1 days relative to index: Magnesium [Mass/volume] in Serum or Plasma (milliequivalent per liter) | 0.054529835 |
| 3.0165E+12 | measurement value during day -365 through -1 days relative to index: Oxygen saturation in Arterial blood (percent) | 0.049949944 |
| 4086687210 | condition_era group during day -365 through -1 days relative to index: Lump on extremities | 0.049935137 |
| 4140090210 | condition_era group during day -365 through -1 days relative to index: Parkinsonism | 0.049755747 |
| 986417 | drug_era only per oral during day -365 through -1 days relative to index: polyethylene glycol 3350 | 0.049440301 |
| 40492458102 | condition_occurrence during day -365 through -1 days relative to index: Neoplasm of uncertain behavior of digestive organ | 0.049136225 |
| 42538830210 | condition_era group during day -365 through -1 days relative to index: Abnormal blood cell count | 0.04820533 |
| 4308811210 | condition_era group during day -365 through -1 days relative to index: Neoplasm of soft tissue | 0.045036184 |
| 2.001E+15 | measurement value during day -365 through -1 days relative to index: RF211_right Total T-score (score) | 0.043962888 |
| 1139042 | drug_era only per oral during day -365 through -1 days relative to index: acetylcysteine | 0.042512378 |
| 199866210 | condition_era group during day -365 through -1 days relative to index: Acute gastritis | 0.042462547 |
| 134736102 | condition_occurrence during day -365 through -1 days relative to index: Backache | 0.040744828 |
| 4103523502 | procedure_occurrence during day -365 through -1 days relative to index: Laryngoscopy | 0.040282527 |
| 443258210 | condition_era group during day -365 through -1 days relative to index: Mass in head or neck | 0.039695167 |
| 200452102 | condition_occurrence during day -365 through -1 days relative to index: Disorder of female genital organs | 0.039407715 |
| 1195334 | drug_era only per oral during day -365 through -1 days relative to index: choline | 0.039207581 |
| 3.00209E+12 | measurement value during day -365 through -1 days relative to index: Forced vital capacity [Volume] Respiratory system Predicted (liter) | 0.036308271 |
| 40492458210 | condition_era group during day -365 through -1 days relative to index: Neoplasm of uncertain behavior of digestive organ | 0.035764858 |
| 4302654210 | condition_era group during day -365 through -1 days relative to index: Itching | 0.035694761 |
| 1550557 | drug_era only per oral during day -365 through -1 days relative to index: prednisolone | 0.035253477 |
| 4051221210 | condition_era group during day -365 through -1 days relative to index: Increased lipid | 0.03302619 |
| 4133611210 | condition_era group during day -365 through -1 days relative to index: Traumatic AND/OR non-traumatic brain injury | 0.032176364 |
| 43009023 | drug_era only per oral during day -365 through -1 days relative to index: levosulpiride | 0.03214119 |
| 252280210 | condition_era group during day -365 through -1 days relative to index: Neoplasm of respiratory tract | 0.031869557 |
| 201826102 | condition_occurrence during day -365 through -1 days relative to index: Type 2 diabetes mellitus | 0.030809892 |
| 36713289502 | procedure_occurrence during day -365 through -1 days relative to index: CT angiography of head | 0.030007728 |
| 3.0119E+12 | measurement value during day -365 through -1 days relative to index: Phosphate [Mass/volume] in Serum or Plasma (milligram per deciliter) | 0.028782955 |
| 432582210 | condition_era group during day -365 through -1 days relative to index: Neoplastic disease of uncertain behavior | 0.028691037 |
| 4083787210 | condition_era group during day -365 through -1 days relative to index: Skin or mucosa lesion | 0.026878657 |
| 916005 | drug_era only per oral during day -365 through -1 days relative to index: solifenacin | 0.026378749 |
| 19095164 | drug_era only per oral during day -365 through -1 days relative to index: cholecalciferol | 0.025862508 |
| 4291649210 | condition_era group during day -365 through -1 days relative to index: Upper gastrointestinal bleeding | 0.025859611 |
| 439847210 | condition_era group during day -365 through -1 days relative to index: Intracranial hemorrhage | 0.024128555 |
| 2E+15 | measurement value during day -365 through -1 days relative to index: Indicator of Surgery Cancer (score) | 0.023825906 |
| 3.03512E+12 | measurement value during day -365 through -1 days relative to index: Erythrocytes [#/area] in Urine sediment by Microscopy high power field (per high power field) | 0.023782529 |
| 19049105 | drug_era only per oral during day -365 through -1 days relative to index: potassium chloride | 0.023596645 |
| 1328165 | drug_era only per oral during day -365 through -1 days relative to index: diltiazem | 0.023511381 |
| 929887 | drug_era only per oral during day -365 through -1 days relative to index: lansoprazole | 0.022096873 |
| 46272801502 | procedure_occurrence during day -365 through -1 days relative to index: CT of head with contrast | 0.02120235 |
| 1716903 | drug_era only per oral during day -365 through -1 days relative to index: moxifloxacin | 0.020926475 |
| 440921210 | condition_era group during day -365 through -1 days relative to index: Traumatic injury | 0.020453842 |
| 43009043 | drug_era only per oral during day -365 through -1 days relative to index: camostat mesilate | 0.019908809 |
| 4338120210 | condition_era group during day -365 through -1 days relative to index: Altered bowel function | 0.018982553 |
| 4339468210 | condition_era group during day -365 through -1 days relative to index: Ear, nose and throat disorder | 0.016761009 |
| 3.03558E+12 | measurement value during day -365 through -1 days relative to index: Leukocytes [#/area] in Urine sediment by Microscopy high power field (per high power field) | 0.016650874 |
| 3.00691E+12 | measurement value during day -365 through -1 days relative to index: Calcium [Mass/volume] in Serum or Plasma (milligram per deciliter) | 0.015210364 |
| 1341238 | drug_era only per oral during day -365 through -1 days relative to index: terazosin | 0.013978381 |
| 4110575102 | condition_occurrence during day -365 through -1 days relative to index: Adenocarcinoma of rectum | 0.012662933 |
| 4310408502 | procedure_occurrence during day -365 through -1 days relative to index: Distal subtotal gastrectomy | 0.011067417 |
| 441408210 | condition_era group during day -365 through -1 days relative to index: Vomiting | 0.009695959 |
| 1337620 | drug_era only per oral during day -365 through -1 days relative to index: capecitabine | 0.00886637 |
| 3.02063E+12 | measurement value during day -365 through -1 days relative to index: Protein [Mass/volume] in Serum or Plasma (gram per deciliter) | 0.008068082 |
| 1735947 | drug_era only per oral during day -365 through -1 days relative to index: rifaximin | 0.007894725 |
| 4048027210 | condition_era group during day -365 through -1 days relative to index: Neuropathy associated with endocrine disorder | 0.007601577 |
| 4306317502 | procedure_occurrence during day -365 through -1 days relative to index: CT angiography of coronary arteries | 0.007401583 |
| 4203711802 | observation during day -365 through -1 days relative to index: Follow-up status | 0.007397083 |
| 3.00334E+12 | measurement value during day -365 through -1 days relative to index: MCHC [Mass/volume] (gram per deciliter) | 0.006991852 |
| 4318377210 | condition_era group during day -365 through -1 days relative to index: Mucocutaneous ulcer | 0.006730488 |
| 4223530210 | condition_era group during day -365 through -1 days relative to index: Spondylitis | 0.005792799 |
| 1503297 | drug_era only per oral during day -365 through -1 days relative to index: metformin | 0.004841555 |
| 139750210 | condition_era group during day -365 through -1 days relative to index: Primary malignant neoplasm of skin | 0.004405429 |
| 961047 | drug_era only per oral during day -365 through -1 days relative to index: ranitidine | 0.001893891 |
| 37117305502 | procedure_occurrence during day -365 through -1 days relative to index: CT of abdomen and pelvis without contrast | 0.001538345 |
| 3.00479E+12 | measurement value during day -365 through -1 days relative to index: Transferrin [Mass/volume] in Serum or Plasma (milligram per deciliter) | 0.001454373 |
| 42898675 | drug_era only per oral during day -365 through -1 days relative to index: Bacillus subtilis | 0.000969733 |
| 1750500 | drug_era only per oral during day -365 through -1 days relative to index: clarithromycin | 0.000837032 |
| 4154801210 | condition_era group during day -365 through -1 days relative to index: Observation of sensation | 0.00054402 |
| 432867210 | condition_era group during day -365 through -1 days relative to index: Hyperlipidemia | 0.000297003 |
| 4098954102 | condition_occurrence during day -365 through -1 days relative to index: Klatskin's tumor | 5.84E-05 |
| 321314210 | condition_era group during day -365 through -1 days relative to index: Abdominal aortic aneurysm without rupture | 3.72E-08 |
| 4098954210 | condition_era group during day -365 through -1 days relative to index: Klatskin's tumor | 1.85E-09 |
| 4300243210 | condition_era group during day -365 through -1 days relative to index: Postoperative complication | -1.13E-05 |
| 44788734210 | condition_era group during day -365 through -1 days relative to index: Complaining of weight loss | -4.94E-05 |
| 4087381502 | procedure_occurrence during day -365 through -1 days relative to index: Sigmoidoscopy | -0.000770487 |
| 1580747 | drug_era only per oral during day -365 through -1 days relative to index: sitagliptin | -0.000812534 |
| 1103314 | drug_era only per oral during day -365 through -1 days relative to index: tramadol | -0.001110191 |
| 436096210 | condition_era group during day -365 through -1 days relative to index: Chronic pain | -0.001385968 |
| 4129880210 | condition_era group during day -365 through -1 days relative to index: Neoplasm of sigmoid colon | -0.00157114 |
| 440649102 | condition_occurrence during day -365 through -1 days relative to index: Primary malignant neoplasm of head of pancreas | -0.001641588 |
| 4162369210 | condition_era group during day -365 through -1 days relative to index: Liquid stool | -0.002740656 |
| 43009021 | drug_era only per oral during day -365 through -1 days relative to index: iron acetyl transferrin | -0.00363949 |
| 4263848210 | condition_era group during day -365 through -1 days relative to index: Dyspnea on exertion | -0.005340034 |
| 1713332 | drug_era only per oral during day -365 through -1 days relative to index: amoxicillin | -0.006151566 |
| 4214956802 | observation during day -365 through -1 days relative to index: History of clinical finding in subject | -0.007862347 |
| 192359210 | condition_era group during day -365 through -1 days relative to index: Renal failure syndrome | -0.007891197 |
| 196931210 | condition_era group during day -365 through -1 days relative to index: Neoplasm of digestive tract | -0.008808298 |
| 77079210 | condition_era group during day -365 through -1 days relative to index: Spinal stenosis | -0.009764977 |
| 195482210 | condition_era group during day -365 through -1 days relative to index: Primary malignant neoplasm of intestinal tract | -0.010208902 |
| 19136187 | drug_era only per oral during day -365 through -1 days relative to index: streptokinase | -0.011728046 |
| 73553210 | condition_era group during day -365 through -1 days relative to index: Arthropathy | -0.012440721 |
| 4239733210 | condition_era group during day -365 through -1 days relative to index: Benign neoplasm of biliary tract | -0.012979258 |
| 441561210 | condition_era group during day -365 through -1 days relative to index: Low tension glaucoma | -0.013123635 |
| 192671102 | condition_occurrence during day -365 through -1 days relative to index: Gastrointestinal hemorrhage | -0.013706985 |
| 4037322210 | condition_era group during day -365 through -1 days relative to index: C/O: a general symptom | -0.014007274 |
| 4178956210 | condition_era group during day -365 through -1 days relative to index: Vascular disorder of extremity | -0.014100074 |
| 4150129210 | condition_era group during day -365 through -1 days relative to index: Musculoskeletal pain | -0.014201385 |
| 744740 | drug_era only per oral during day -365 through -1 days relative to index: zolpidem | -0.014316023 |
| 443611210 | condition_era group during day -365 through -1 days relative to index: Chronic kidney disease stage 5 | -0.014923286 |
| 45757776210 | condition_era group during day -365 through -1 days relative to index: Abnormal urination | -0.015221845 |
| 198185102 | condition_occurrence during day -365 through -1 days relative to index: Chronic renal failure | -0.015771529 |
| 4247719102 | condition_occurrence during day -365 through -1 days relative to index: Primary malignant neoplasm of ascending colon | -0.017233771 |
| 77670102 | condition_occurrence during day -365 through -1 days relative to index: Chest pain | -0.017754714 |
| 3.02104E+12 | measurement value during day -365 through -1 days relative to index: Iron binding capacity [Mass/volume] in Serum or Plasma (microgram per deciliter) | -0.022448066 |
| 4022173502 | procedure_occurrence during day -365 through -1 days relative to index: Transfusion of red blood cells | -0.023683185 |
| 4169252210 | condition_era group during day -365 through -1 days relative to index: Internal injury of abdominal organ | -0.023899388 |
| 200219210 | condition_era group during day -365 through -1 days relative to index: Abdominal pain | -0.024566734 |
| 443568210 | condition_era group during day -365 through -1 days relative to index: Malignant neoplasm of gastrointestinal tract | -0.027042804 |
| 1769535 | drug_era only per oral during day -365 through -1 days relative to index: cefadroxil | -0.028293653 |
| 19071236 | drug_era only per oral during day -365 through -1 days relative to index: lactitol | -0.02880053 |
| 19041843 | drug_era only per oral during day -365 through -1 days relative to index: carbocysteine | -0.030342881 |
| 4294382502 | procedure_occurrence during day -365 through -1 days relative to index: Esophagogastroduodenoscopy | -0.030739263 |
| 4054503210 | condition_era group during day -365 through -1 days relative to index: Neoplasm of intra-abdominal organs | -0.031287868 |
| 40484102102 | condition_occurrence during day -365 through -1 days relative to index: Abnormal finding on evaluation procedure | -0.032788578 |
| 4144684802 | observation during day -365 through -1 days relative to index: Patient referral | -0.032797366 |
| 4028876210 | condition_era group during day -365 through -1 days relative to index: Inflammatory disorder of lower respiratory tract | -0.034785183 |
| 19058933 | drug_era only per oral during day -365 through -1 days relative to index: erdosteine | -0.034816903 |
| 193782210 | condition_era group during day -365 through -1 days relative to index: End-stage renal disease | -0.034879931 |
| 19011339 | drug_era only per oral during day -365 through -1 days relative to index: mosapride | -0.035803607 |
| 4124706210 | condition_era group during day -365 through -1 days relative to index: Myocardial dysfunction | -0.039003363 |
| 3.00425E+12 | measurement value during day -365 through -1 days relative to index: Systolic blood pressure (millimeter mercury column) | -0.039312196 |
| 36713053502 | procedure_occurrence during day -365 through -1 days relative to index: MR angiography of brain and neck without contrast | -0.04249531 |
| 4113547210 | condition_era group during day -365 through -1 days relative to index: Lesion of stomach | -0.042537878 |
| 3.01725E+12 | measurement value during day -365 through -1 days relative to index: Creatinine [Mass/volume] in Urine (milligram per deciliter) | -0.042944008 |
| 4088035802 | observation during day -365 through -1 days relative to index: Screening status | -0.043353397 |
| 443387102 | condition_occurrence during day -365 through -1 days relative to index: Malignant tumor of stomach | -0.043405988 |
| 432851210 | condition_era group during day -365 through -1 days relative to index: Secondary malignant neoplastic disease | -0.043431881 |
| 4145825210 | condition_era group during day -365 through -1 days relative to index: Anorectal disorder | -0.043874657 |
| 1322184 | drug_era only per oral during day -365 through -1 days relative to index: clopidogrel | -0.04412874 |
| 321588210 | condition_era group during day -365 through -1 days relative to index: Heart disease | -0.04428924 |
| 40239216 | drug_era only per oral during day -365 through -1 days relative to index: linagliptin | -0.044876118 |
| 4208786210 | condition_era group during day -365 through -1 days relative to index: Musculoskeletal and connective tissue disorder | -0.045073111 |
| 4248870210 | condition_era group during day -365 through -1 days relative to index: Visceroptosis | -0.045178351 |
| 4135471502 | procedure_occurrence during day -365 through -1 days relative to index: Radiography of kidney-ureter-bladder | -0.045247079 |
| 4318985210 | condition_era group during day -365 through -1 days relative to index: Degeneration of retina | -0.046377577 |
| 734354 | drug_era only per oral during day -365 through -1 days relative to index: pregabalin | -0.046907155 |
| 4183041102 | condition_occurrence during day -365 through -1 days relative to index: Abdominal discomfort | -0.047217105 |
| 4132552210 | condition_era group during day -365 through -1 days relative to index: Acute digestive system disorder | -0.047272801 |
| 4119777210 | condition_era group during day -365 through -1 days relative to index: Ectactic vein | -0.049715437 |
| 4174763210 | condition_era group during day -365 through -1 days relative to index: Early gastric cancer | -0.05095555 |
| 4087642210 | condition_era group during day -365 through -1 days relative to index: Distention of vein | -0.051469194 |
| 4134440210 | condition_era group during day -365 through -1 days relative to index: Visual system disorder | -0.052162221 |
| 19088167 | drug_era only per oral during day -365 through -1 days relative to index: ambroxol | -0.053203124 |
| 4059290210 | condition_era group during day -365 through -1 days relative to index: Steatosis of liver | -0.053810912 |
| 1734104 | drug_era only per oral during day -365 through -1 days relative to index: azithromycin | -0.054086625 |
| 703547 | drug_era only per oral during day -365 through -1 days relative to index: trazodone | -0.054092011 |
| 3.02425E+12 | measurement value during day -365 through -1 days relative to index: Calcium.ionized [Mass/volume] in Serum or Plasma by Ion-selective membrane electrode (ISE) (millimole per liter) | -0.054408649 |
| 904453 | drug_era only per oral during day -365 through -1 days relative to index: esomeprazole | -0.055255716 |
| 444108210 | condition_era group during day -365 through -1 days relative to index: Finding related to sleep | -0.055397858 |
| 36713361102 | condition_occurrence during day -365 through -1 days relative to index: Primary adenocarcinoma of ascending colon | -0.055509486 |
| 45775324 | drug_era only per oral during day -365 through -1 days relative to index: diatrizoic acid | -0.05867084 |
| 1332418 | drug_era only per oral during day -365 through -1 days relative to index: amlodipine | -0.05871278 |
| 4263848102 | condition_occurrence during day -365 through -1 days relative to index: Dyspnea on exertion | -0.059362299 |
| 192279102 | condition_occurrence during day -365 through -1 days relative to index: Disorder of kidney due to diabetes mellitus | -0.060867125 |
| 44788734102 | condition_occurrence during day -365 through -1 days relative to index: Complaining of weight loss | -0.061520596 |
| 4.35394E+12 | measurement value during day -365 through -1 days relative to index: End tidal carbon dioxide concentration (millimeter mercury column) | -0.062938496 |
| 4133004102 | condition_occurrence during day -365 through -1 days relative to index: Deep venous thrombosis | -0.06486627 |
| 991876 | drug_era only per oral during day -365 through -1 days relative to index: loperamide | -0.067307669 |
| 197500210 | condition_era group during day -365 through -1 days relative to index: Primary malignant neoplasm of colon | -0.068415998 |
| 4178818210 | condition_era group during day -365 through -1 days relative to index: Inflammation of specific body systems | -0.071144279 |
| 953076 | drug_era only per oral during day -365 through -1 days relative to index: famotidine | -0.071561378 |
| 439383210 | condition_era group during day -365 through -1 days relative to index: Vertigo | -0.0718102 |
| 907013 | drug_era only per oral during day -365 through -1 days relative to index: metolazone | -0.072471648 |
| 443432210 | condition_era group during day -365 through -1 days relative to index: Impaired cognition | -0.072746452 |
| 1517070 | drug_era only per oral during day -365 through -1 days relative to index: desmopressin | -0.07332436 |
| 3.02459E+12 | measurement value during day -365 through -1 days relative to index: FEV1/FVC Predicted (percent) | -0.074591159 |
| 4233903210 | condition_era group during day -365 through -1 days relative to index: Mass of thoracic structure | -0.074927342 |
| 442793210 | condition_era group during day -365 through -1 days relative to index: Complication due to diabetes mellitus | -0.076125834 |
| 316866102 | condition_occurrence during day -365 through -1 days relative to index: Hypertensive disorder | -0.0777472 |
| 4083519802 | observation during day -365 through -1 days relative to index: Family history of disorder | -0.078931468 |
| 966991 | drug_era only per oral during day -365 through -1 days relative to index: simethicone | -0.081613102 |
| 2.14906E+13 | measurement value during day -365 through -1 days relative to index: Peak inspiratory flow Respiratory system airway (Unknown unit) | -0.08299423 |
| 133729210 | condition_era group during day -365 through -1 days relative to index: Hyperparathyroidism | -0.085581686 |
| 46271022102 | condition_occurrence during day -365 through -1 days relative to index: Chronic kidney disease | -0.087269756 |
| 4181487210 | condition_era group during day -365 through -1 days relative to index: Malignant tumor of male genital organ | -0.0877198 |
| 4027255210 | condition_era group during day -365 through -1 days relative to index: Structural disorder of heart | -0.090083929 |
| 3.00836E+12 | measurement value during day -365 through -1 days relative to index: Apolipoprotein A-I [Mass/volume] in Serum or Plasma (milligram per deciliter) | -0.091608311 |
| 43009003 | drug_era only per oral during day -365 through -1 days relative to index: lafutidine | -0.092651144 |
| 433595210 | condition_era group during day -365 through -1 days relative to index: Edema | -0.094631999 |
| 948078 | drug_era only per oral during day -365 through -1 days relative to index: pantoprazole | -0.094664148 |
| 442131210 | condition_era group during day -365 through -1 days relative to index: Primary malignant neoplasm of head | -0.106432348 |
| 192956102 | condition_occurrence during day -365 through -1 days relative to index: Cholecystitis | -0.107378397 |
| 4180790102 | condition_occurrence during day -365 through -1 days relative to index: Malignant tumor of colon | -0.107859201 |
| 4256761210 | condition_era group during day -365 through -1 days relative to index: Imaging result abnormal | -0.111380073 |
| 4285898210 | condition_era group during day -365 through -1 days relative to index: Polyp of colon | -0.112652587 |
| 19035704 | drug_era only per oral during day -365 through -1 days relative to index: calcium carbonate | -0.112777837 |
| 443611102 | condition_occurrence during day -365 through -1 days relative to index: Chronic kidney disease stage 5 | -0.113568645 |
| 1154332 | drug_era only per oral during day -365 through -1 days relative to index: pseudoephedrine | -0.113857406 |
| 4134595210 | condition_era group during day -365 through -1 days relative to index: Chronic disease of genitourinary system | -0.114880844 |
| 4267297210 | condition_era group during day -365 through -1 days relative to index: Acute cholangitis | -0.115808343 |
| 1201620 | drug_era only per oral during day -365 through -1 days relative to index: codeine | -0.116379236 |
| 42538046210 | condition_era group during day -365 through -1 days relative to index: Perioperative complication | -0.118479351 |
| 4094202210 | condition_era group during day -365 through -1 days relative to index: Space-occupying lesion of nervous system | -0.118927388 |
| 4194610210 | condition_era group during day -365 through -1 days relative to index: Trunk arterial embolus | -0.120372738 |
| 3.01518E+12 | measurement value during day -365 through -1 days relative to index: Erythrocyte distribution width [Entitic volume] by Automated count (femtoliter) | -0.120540939 |
| 192438210 | condition_era group during day -365 through -1 days relative to index: Abdominal mass | -0.121335953 |
| 4182572802 | observation during day -365 through -1 days relative to index: Radiotherapy to abdomen | -0.126893549 |
| 4109085210 | condition_era group during day -365 through -1 days relative to index: Right sided abdominal pain | -0.127254107 |
| 4091134502 | procedure_occurrence during day -365 through -1 days relative to index: Pure tone audiometry | -0.12782641 |
| 2.14909E+13 | measurement value during day -365 through -1 days relative to index: Invasive Systolic blood pressure (millimeter mercury column) | -0.129247429 |
| 441969102 | condition_occurrence during day -365 through -1 days relative to index: Radiology result abnormal | -0.130762189 |
| 4306292210 | condition_era group during day -365 through -1 days relative to index: Upper abdominal pain | -0.130902906 |
| 192438102 | condition_occurrence during day -365 through -1 days relative to index: Abdominal mass | -0.131011413 |
| 433435210 | condition_era group during day -365 through -1 days relative to index: Carcinoma in situ | -0.13320137 |
| 1167322 | drug_era only per oral during day -365 through -1 days relative to index: allopurinol | -0.135320573 |
| 374384210 | condition_era group during day -365 through -1 days relative to index: Cerebral ischemia | -0.136011982 |
| 4310400102 | condition_occurrence during day -365 through -1 days relative to index: Acute appendicitis | -0.137953805 |
| 4249893502 | procedure_occurrence during day -365 through -1 days relative to index: Colonoscopy | -0.138020392 |
| 441561102 | condition_occurrence during day -365 through -1 days relative to index: Low tension glaucoma | -0.139353864 |
| 4036803502 | procedure_occurrence during day -365 through -1 days relative to index: General examination of patient | -0.142431055 |
| 196456102 | condition_occurrence during day -365 through -1 days relative to index: Gallstone | -0.145697314 |
| 4302836210 | condition_era group during day -365 through -1 days relative to index: Neoplasm of endocrine gland | -0.148060108 |
| 8532001 | gender = FEMALE | -0.150602073 |
| 372448210 | condition_era group during day -365 through -1 days relative to index: Loss of consciousness | -0.151467821 |
| 4151121502 | procedure_occurrence during day -365 through -1 days relative to index: Low anterior resection of rectum | -0.151925604 |
| 4201717210 | condition_era group during day -365 through -1 days relative to index: Ileostomy present | -0.152028259 |
| 952004 | drug_era only per oral during day -365 through -1 days relative to index: sevelamer | -0.153948786 |
| 1759842 | drug_era only per oral during day -365 through -1 days relative to index: clavulanate | -0.153960343 |
| 4134294210 | condition_era group during day -365 through -1 days relative to index: Acute inflammatory disease | -0.154150533 |
| 3.02008E+12 | measurement value during day -365 through -1 days relative to index: FVC pre bronchodilation measured/predicted (percent) | -0.155292894 |
| 432257102 | condition_occurrence during day -365 through -1 days relative to index: Primary malignant neoplasm of transverse colon | -0.157679665 |
| 4304943502 | procedure_occurrence during day -365 through -1 days relative to index: Endoscopic retrograde cholangiopancreatography | -0.159177025 |
| 312349210 | condition_era group during day -365 through -1 days relative to index: Venous varices | -0.163197494 |
| 438112210 | condition_era group during day -365 through -1 days relative to index: Neoplastic disease | -0.170987361 |
| 4102111102 | condition_occurrence during day -365 through -1 days relative to index: Mass of body structure | -0.177116265 |
| 781039 | drug_era only per oral during day -365 through -1 days relative to index: alprazolam | -0.177144351 |
| 3.0067E+12 | measurement value during day -365 through -1 days relative to index: P wave axis (Unknown unit) | -0.177350956 |
| 951469 | drug_era only per oral during day -365 through -1 days relative to index: calcium acetate | -0.177387733 |
| 4101227210 | condition_era group during day -365 through -1 days relative to index: Liver mass | -0.17750065 |
| 435506210 | condition_era group during day -365 through -1 days relative to index: Benign neoplastic disease | -0.181193636 |
| 4148972502 | procedure_occurrence during day -365 through -1 days relative to index: Extubation of trachea | -0.184286943 |
| 4145627210 | condition_era group during day -365 through -1 days relative to index: Biliary calculus | -0.184996691 |
| 4028367210 | condition_era group during day -365 through -1 days relative to index: Acute disease of cardiovascular system | -0.187465742 |
| 43525922 | drug_era only per oral during day -365 through -1 days relative to index: Coptis chinensis root extract | -0.188247744 |
| 40479817802 | observation during day -365 through -1 days relative to index: Hospital falls risk assessment score for the elderly | -0.18971993 |
| 4046549802 | observation during day -365 through -1 days relative to index: Preoperative care | -0.190848915 |
| 4022830210 | condition_era group during day -365 through -1 days relative to index: General problem AND/OR complaint | -0.1946308 |
| 3.02202E+12 | measurement value during day -365 through -1 days relative to index: QRS duration (Unknown unit) | -0.196673328 |
| 440358210 | condition_era group during day -365 through -1 days relative to index: Lipoma (clinical) | -0.196698915 |
| 3.02581E+12 | measurement value during day -365 through -1 days relative to index: Q-T interval (Unknown unit) | -0.199436741 |
| 970250 | drug_era only per oral during day -365 through -1 days relative to index: spironolactone | -0.206364953 |
| 3.01034E+12 | measurement value during day -365 through -1 days relative to index: Triiodothyronine (T3) [Mass/volume] in Serum or Plasma (nanogram per deciliter) | -0.206724731 |
| 4101343102 | condition_occurrence during day -365 through -1 days relative to index: Functional finding of gastrointestinal tract | -0.20896092 |
| 199754102 | condition_occurrence during day -365 through -1 days relative to index: Primary malignant neoplasm of pancreas | -0.214565052 |
| 4104000210 | condition_era group during day -365 through -1 days relative to index: Lesion of liver | -0.216230578 |
| 436635102 | condition_occurrence during day -365 through -1 days relative to index: Primary malignant neoplasm of sigmoid colon | -0.217592441 |
| 1784749 | drug_era only per oral during day -365 through -1 days relative to index: kanamycin | -0.224508802 |
| 439777102 | condition_occurrence during day -365 through -1 days relative to index: Anemia | -0.227558598 |
| 40486024210 | condition_era group during day -365 through -1 days relative to index: Cyst of abdomen | -0.232563934 |
| 19011331 | drug_era only per oral during day -365 through -1 days relative to index: barium sulfate | -0.234502834 |
| 40480615210 | condition_era group during day -365 through -1 days relative to index: Cognitive disorder | -0.24028107 |
| 19136184 | drug_era only per oral during day -365 through -1 days relative to index: streptodornase | -0.244728898 |
| 3.03628E+12 | measurement value during day -365 through -1 days relative to index: Body height (centimeter) | -0.246739461 |
| 444205210 | condition_era group during day -365 through -1 days relative to index: Post-viral disorder | -0.247468295 |
| 4030518210 | condition_era group during day -365 through -1 days relative to index: Renal impairment | -0.255638989 |
| 4022013502 | procedure_occurrence during day -365 through -1 days relative to index: Pylorus-sparing Whipple operation | -0.260069463 |
| 4130373210 | condition_era group during day -365 through -1 days relative to index: Neoplasm of gastrointestinal tract | -0.261999396 |
| 4110707210 | condition_era group during day -365 through -1 days relative to index: Epithelial neoplasm of skin | -0.262002613 |
| 4051104802 | observation during day -365 through -1 days relative to index: No family history of | -0.263065431 |
| 3.01289E+12 | measurement value during day -365 through -1 days relative to index: Diastolic blood pressure (millimeter mercury column) | -0.263659411 |
| 939871 | drug_era only per oral during day -365 through -1 days relative to index: sodium phosphate | -0.273165218 |
| 4028373210 | condition_era group during day -365 through -1 days relative to index: Hernia of abdominal wall | -0.274301399 |
| 4103703210 | condition_era group during day -365 through -1 days relative to index: Melena | -0.290165541 |
| 3.03751E+12 | measurement value during day -365 through -1 days relative to index: Lymphocytes/100 leukocytes in Blood by Automated count (percent) | -0.294164786 |
| 40484156210 | condition_era group during day -365 through -1 days relative to index: Malignant adenomatous neoplasm | -0.303477169 |
| 3.04311E+12 | measurement value during day -365 through -1 days relative to index: Platelet mean volume [Entitic volume] in Blood by Automated count (femtoliter) | -0.30444613 |
| 939506 | drug_era only per oral during day -365 through -1 days relative to index: sodium bicarbonate | -0.308560278 |
| 195856210 | condition_era group during day -365 through -1 days relative to index: Cholangitis | -0.314478723 |
| 37117806502 | procedure_occurrence during day -365 through -1 days relative to index: MRI of bilateral breasts with contrast | -0.315589564 |
| 3.02331E+12 | measurement value during day -365 through -1 days relative to index: Hematocrit [Volume Fraction] of Blood by Automated count (percent) | -0.332659873 |
| 198809102 | condition_occurrence during day -365 through -1 days relative to index: Acute cholecystitis | -0.353100724 |
| 45757776102 | condition_occurrence during day -365 through -1 days relative to index: Abnormal urination | -0.354850473 |
| 4174763102 | condition_occurrence during day -365 through -1 days relative to index: Early gastric cancer | -0.36895123 |
| 43009007 | drug_era only per oral during day -365 through -1 days relative to index: polmacoxib | -0.384169661 |
| 2E+15 | measurement value during day -365 through -1 days relative to index: PEF pre bronchodilation measured/predicted (percent) | -0.392071987 |
| 4235749502 | procedure_occurrence during day -365 through -1 days relative to index: Laparoscopic-assisted anterior resection of rectum | -0.392626343 |
| 443530102 | condition_occurrence during day -365 through -1 days relative to index: Hematochezia | -0.401910397 |
| 4273629502 | procedure_occurrence during day -365 through -1 days relative to index: Chemotherapy | -0.409767445 |
| 192956210 | condition_era group during day -365 through -1 days relative to index: Cholecystitis | -0.421735435 |
| 198464210 | condition_era group during day -365 through -1 days relative to index: Incisional hernia | -0.422501366 |
| 4103992210 | condition_era group during day -365 through -1 days relative to index: Mass of stomach | -0.44555169 |
| 199860210 | condition_era group during day -365 through -1 days relative to index: Hernia of abdominal cavity | -0.455290402 |
| 3.03366E+12 | measurement value during day -365 through -1 days relative to index: Prothrombin time (PT) actual/Normal (percent) | -0.468489472 |
| 135772210 | condition_era group during day -365 through -1 days relative to index: Goiter | -0.49179995 |
| 40488439210 | condition_era group during day -365 through -1 days relative to index: Abnormality of systemic vein | -0.529889901 |
| 4115576210 | condition_era group during day -365 through -1 days relative to index: Lesion of gallbladder | -0.549819349 |
| 141253102 | condition_occurrence during day -365 through -1 days relative to index: Disorder of thyroid gland | -0.555932292 |
| 3.00655E+12 | measurement value during day -365 through -1 days relative to index: Fluid intake oral Estimated (Non-specific) | -0.565181618 |
| 81251210 | condition_era group during day -365 through -1 days relative to index: Neoplasm of breast | -0.56915906 |
| 35622827502 | procedure_occurrence during day -365 through -1 days relative to index: Radionuclide imaging of liver and/or biliary tract using radioactive isotope | -0.599896175 |
| 4.04838E+13 | measurement value during day -365 through -1 days relative to index: Total radiation dose delivered (centigray) | -0.682322345 |
| 4196958502 | procedure_occurrence during day -365 through -1 days relative to index: Laparoscopic procedure | -0.694717804 |
| 440448210 | condition_era group during day -365 through -1 days relative to index: Appendicitis | -0.709953602 |
| 4201717102 | condition_occurrence during day -365 through -1 days relative to index: Ileostomy present | -0.769081936 |
| 45765544502 | procedure_occurrence during day -365 through -1 days relative to index: CT of thyroid with contrast | -0.834803762 |
| 4288544210 | condition_era group during day -365 through -1 days relative to index: Inguinal hernia | -1.393154809 |
| 3.02456E+12 | measurement value during day -365 through -1 days relative to index: Albumin [Mass/volume] in Serum or Plasma (gram per deciliter) | -2.695270417 |
