# Supplementary material for: Development and Validation of a Prognostic Classification Model Predicting Postoperative Adverse Outcomes in Older Surgical Patients Using a Machine Learning Algorithm: Retrospective Observational Network Study
Source: J Med Internet Res. 2023 Nov 13;25:e42259. doi: 10.2196/42259 (PMC10682929; doi:10.2196/42259)
Supplement: Multimedia Appendix 10 [file jmir_v25i1e42259_app10.docx]

Selected 237 variables and covariate values of the Lasso Logistic Regression model for prolonged hospital stay

| covariateId | covariateName | covariateValue |
| --- | --- | --- |
| 3.02413E+12 | measurement value during day -365 through -1 days relative to index: Bilirubin.total [Mass/volume] in Serum or Plasma (milligram per deciliter) | 1.363415816 |
| 4167096210 | condition_era group during day -365 through -1 days relative to index: Osteitis | 0.83376741 |
| 3.02046E+12 | measurement value during day -365 through -1 days relative to index: C reactive protein [Mass/volume] in Serum or Plasma (milligram per deciliter) | 0.624536681 |
| 3.01261E+12 | measurement value during day -365 through -1 days relative to index: Segmented neutrophils/100 leukocytes in Blood by Automated count (percent) | 0.620362276 |
| 4143397210 | condition_era group during day -365 through -1 days relative to index: Mass of pancreas | 0.619365612 |
| 4193176210 | condition_era group during day -365 through -1 days relative to index: Musculoskeletal infective disorder | 0.539228501 |
| 4129884210 | condition_era group during day -365 through -1 days relative to index: Neoplasm of biliary tract | 0.502774 |
| 36712821210 | condition_era group during day -365 through -1 days relative to index: Postprocedural infection | 0.482055589 |
| 436222102 | condition_occurrence during day -365 through -1 days relative to index: Altered mental status | 0.411674985 |
| 967861 | drug_era only per oral during day -365 through -1 days relative to index: magnesium citrate | 0.410769227 |
| 4262920210 | condition_era group during day -365 through -1 days relative to index: Skin ulcer | 0.379708705 |
| 4041681210 | condition_era group during day -365 through -1 days relative to index: System disorder of the nervous system | 0.373105392 |
| 1300978 | drug_era only per oral during day -365 through -1 days relative to index: megestrol | 0.363964307 |
| 4302654210 | condition_era group during day -365 through -1 days relative to index: Itching | 0.357113243 |
| 4161393502 | procedure_occurrence during day -365 through -1 days relative to index: MRI of pelvis with contrast | 0.347499863 |
| 312336210 | condition_era group during day -365 through -1 days relative to index: Arterial aneurysm | 0.329559029 |
| 3.01641E+12 | measurement value during day -365 through -1 days relative to index: Fibrinogen [Mass/volume] in Platelet poor plasma by Coagulation assay (milligram per deciliter) | 0.328567405 |
| 1796458 | drug_era only per oral during day -365 through -1 days relative to index: cefdinir | 0.318863623 |
| 1786842 | drug_era only per oral during day -365 through -1 days relative to index: cephradine | 0.316124432 |
| 4047650210 | condition_era group during day -365 through -1 days relative to index: Neoplasm of ovary | 0.29624434 |
| 1124957 | drug_era only per oral during day -365 through -1 days relative to index: oxycodone | 0.295155493 |
| 252280210 | condition_era group during day -365 through -1 days relative to index: Neoplasm of respiratory tract | 0.291837663 |
| 36713285502 | procedure_occurrence during day -365 through -1 days relative to index: CT of lumbar spine without contrast | 0.291464598 |
| 4095434210 | condition_era group during day -365 through -1 days relative to index: Malignant neoplasm of hepatic duct | 0.282180027 |
| 4.28685E+13 | measurement value during day -365 through -1 days relative to index: FEV1/FVC --post bronchodilation (percent) | 0.278074563 |
| 434621210 | condition_era group during day -365 through -1 days relative to index: Autoimmune disease | 0.27532955 |
| 3.00425E+12 | measurement value during day -365 through -1 days relative to index: Systolic blood pressure (millimeter mercury column) | 0.268983207 |
| 3.0199E+12 | measurement value during day -365 through -1 days relative to index: Erythrocyte distribution width [Ratio] by Automated count (percent) | 0.261638106 |
| 321318102 | condition_occurrence during day -365 through -1 days relative to index: Angina pectoris | 0.238086801 |
| 4244986502 | procedure_occurrence during day -365 through -1 days relative to index: CT of brain without contrast | 0.234756699 |
| 4.07738E+13 | measurement value during day -365 through -1 days relative to index: Erythrocyte Distribution Width \| Red Blood Cells (percent) | 0.22997464 |
| 19059796 | drug_era only per oral during day -365 through -1 days relative to index: gliclazide | 0.216678349 |
| 138525210 | condition_era group during day -365 through -1 days relative to index: Pain in limb | 0.214565425 |
| 3.0276E+12 | measurement value during day -365 through -1 days relative to index: Mean blood pressure (millimeter mercury column) | 0.212572018 |
| 77079210 | condition_era group during day -365 through -1 days relative to index: Spinal stenosis | 0.212313088 |
| 439847210 | condition_era group during day -365 through -1 days relative to index: Intracranial hemorrhage | 0.210195163 |
| 4068261210 | condition_era group during day -365 through -1 days relative to index: Bile duct proliferation | 0.206160762 |
| 45765485502 | procedure_occurrence during day -365 through -1 days relative to index: CT angiography of neck vessels | 0.202823364 |
| 200680210 | condition_era group during day -365 through -1 days relative to index: Neoplasm of bladder | 0.200833528 |
| 2.001E+15 | measurement value during day -365 through -1 days relative to index: RF211_right Total T-score (score) | 0.198899521 |
| 1749008 | drug_era only per oral during day -365 through -1 days relative to index: cefpodoxime | 0.18855089 |
| 4154630210 | condition_era group during day -365 through -1 days relative to index: Malignant neoplasm of genitourinary organ | 0.18490368 |
| 4113545210 | condition_era group during day -365 through -1 days relative to index: Lesion of esophagus | 0.184344266 |
| 40485387210 | condition_era group during day -365 through -1 days relative to index: Neoplasm of skeletal system | 0.17733712 |
| 1118084 | drug_era only per oral during day -365 through -1 days relative to index: celecoxib | 0.176395237 |
| 373718210 | condition_era group during day -365 through -1 days relative to index: Neoplasm of connective tissues | 0.157208214 |
| 1114220 | drug_era only per oral during day -365 through -1 days relative to index: naloxone | 0.153676017 |
| 4099350210 | condition_era group during day -365 through -1 days relative to index: Infectious disease of genitourinary system | 0.151335776 |
| 3.02089E+12 | measurement value during day -365 through -1 days relative to index: Body temperature (degree Celsius) | 0.143394417 |
| 4058335502 | procedure_occurrence during day -365 through -1 days relative to index: CT of chest | 0.141614328 |
| 4248028210 | condition_era group during day -365 through -1 days relative to index: Supraventricular arrhythmia | 0.14158162 |
| 906780 | drug_era only per oral during day -365 through -1 days relative to index: metoclopramide | 0.140357458 |
| 4129869210 | condition_era group during day -365 through -1 days relative to index: Neoplasm of soft tissues of abdomen | 0.139539625 |
| 40491001210 | condition_era group during day -365 through -1 days relative to index: Malignant neoplasm of digestive system | 0.137275845 |
| 439392210 | condition_era group during day -365 through -1 days relative to index: Primary malignant neoplasm | 0.131240913 |
| 4087641210 | condition_era group during day -365 through -1 days relative to index: Distention of artery | 0.129646277 |
| 1195334 | drug_era only per oral during day -365 through -1 days relative to index: choline | 0.128479851 |
| 4129389210 | condition_era group during day -365 through -1 days relative to index: Gastrointestinal obstruction | 0.127022198 |
| 79908210 | condition_era group during day -365 through -1 days relative to index: Muscle weakness | 0.125711394 |
| 437312210 | condition_era group during day -365 through -1 days relative to index: Bleeding | 0.122388964 |
| 4203711802 | observation during day -365 through -1 days relative to index: Follow-up status | 0.120868343 |
| 134736210 | condition_era group during day -365 through -1 days relative to index: Backache | 0.117074119 |
| 904453 | drug_era only per oral during day -365 through -1 days relative to index: esomeprazole | 0.11002594 |
| 444187210 | condition_era group during day -365 through -1 days relative to index: Open wound | 0.109797023 |
| 1318853 | drug_era only per oral during day -365 through -1 days relative to index: nifedipine | 0.10916955 |
| 1707164 | drug_era only per oral during day -365 through -1 days relative to index: metronidazole | 0.107823494 |
| 201826102 | condition_occurrence during day -365 through -1 days relative to index: Type 2 diabetes mellitus | 0.107105038 |
| 19049105 | drug_era only per oral during day -365 through -1 days relative to index: potassium chloride | 0.106646078 |
| 4181345210 | condition_era group during day -365 through -1 days relative to index: Malignant tumor of biliary tract | 0.104312006 |
| 4022173502 | procedure_occurrence during day -365 through -1 days relative to index: Transfusion of red blood cells | 0.101253904 |
| 440921210 | condition_era group during day -365 through -1 days relative to index: Traumatic injury | 0.101218195 |
| 4214956802 | observation during day -365 through -1 days relative to index: History of clinical finding in subject | 0.100066881 |
| 317585210 | condition_era group during day -365 through -1 days relative to index: Aortic aneurysm | 0.098787749 |
| 4090425210 | condition_era group during day -365 through -1 days relative to index: Altered sensation of skin | 0.093845368 |
| 987245 | drug_era only per oral during day -365 through -1 days relative to index: lactulose | 0.093112314 |
| 4217238502 | procedure_occurrence during day -365 through -1 days relative to index: Radioisotope scan of bone | 0.089288713 |
| 4176946210 | condition_era group during day -365 through -1 days relative to index: Inflammatory disorder of musculoskeletal system | 0.088836054 |
| 80809210 | condition_era group during day -365 through -1 days relative to index: Rheumatoid arthritis | 0.087934592 |
| 766814 | drug_era only per oral during day -365 through -1 days relative to index: quetiapine | 0.083429434 |
| 1560524 | drug_era only per oral during day -365 through -1 days relative to index: glucose | 0.074222199 |
| 4051104802 | observation during day -365 through -1 days relative to index: No family history of | 0.073330222 |
| 43021868210 | condition_era group during day -365 through -1 days relative to index: Aneurysm of peripheral artery | 0.07302841 |
| 4029305102 | condition_occurrence during day -365 through -1 days relative to index: Hypercholesterolemia | 0.07041454 |
| 36879181 | drug_era only per oral during day -365 through -1 days relative to index: streptococcus faecalis | 0.068708771 |
| 3.0165E+12 | measurement value during day -365 through -1 days relative to index: Oxygen saturation in Arterial blood (percent) | 0.067605234 |
| 4042056210 | condition_era group during day -365 through -1 days relative to index: Lipids abnormal | 0.067069931 |
| 43009053 | drug_era only per oral during day -365 through -1 days relative to index: itopride hydrochloride | 0.063131021 |
| 197304210 | condition_era group during day -365 through -1 days relative to index: Ulcer of lower extremity | 0.061405666 |
| 1036228 | drug_era only per oral during day -365 through -1 days relative to index: sucralfate | 0.060397022 |
| 40481925802 | observation during day -365 through -1 days relative to index: No history of clinical finding in subject | 0.058634582 |
| 1503297 | drug_era only per oral during day -365 through -1 days relative to index: metformin | 0.05802963 |
| 777221 | drug_era only per oral during day -365 through -1 days relative to index: hydroxyzine | 0.057332409 |
| 1346823 | drug_era only per oral during day -365 through -1 days relative to index: carvedilol | 0.051391264 |
| 432867210 | condition_era group during day -365 through -1 days relative to index: Hyperlipidemia | 0.050389722 |
| 197506210 | condition_era group during day -365 through -1 days relative to index: Malignant neoplasm of abdomen | 0.049673943 |
| 443784210 | condition_era group during day -365 through -1 days relative to index: Vascular disorder | 0.048370548 |
| 1550557 | drug_era only per oral during day -365 through -1 days relative to index: prednisolone | 0.04791809 |
| 4179094210 | condition_era group during day -365 through -1 days relative to index: Malignant tumor of soft tissue of abdomen | 0.039896565 |
| 4181343102 | condition_occurrence during day -365 through -1 days relative to index: Malignant tumor of esophagus | 0.037149066 |
| 312437210 | condition_era group during day -365 through -1 days relative to index: Dyspnea | 0.03656717 |
| 1112807 | drug_era only per oral during day -365 through -1 days relative to index: aspirin | 0.03409245 |
| 45765716502 | procedure_occurrence during day -365 through -1 days relative to index: MRI of head and neck with contrast | 0.033785088 |
| 4335825502 | procedure_occurrence during day -365 through -1 days relative to index: Transthoracic echocardiography | 0.033515924 |
| 1153428 | drug_era only per oral during day -365 through -1 days relative to index: fexofenadine | 0.030356839 |
| 4316083210 | condition_era group during day -365 through -1 days relative to index: Skin lesion | 0.026090408 |
| 929887 | drug_era only per oral during day -365 through -1 days relative to index: lansoprazole | 0.025733981 |
| 4324347502 | procedure_occurrence during day -365 through -1 days relative to index: CT of liver with contrast | 0.02500387 |
| 31317102 | condition_occurrence during day -365 through -1 days relative to index: Dysphagia | 0.024206389 |
| 3.02104E+12 | measurement value during day -365 through -1 days relative to index: Iron binding capacity [Mass/volume] in Serum or Plasma (microgram per deciliter) | 0.02395874 |
| 443386210 | condition_era group during day -365 through -1 days relative to index: Malignant tumor of duodenum | 0.02381355 |
| 1139042 | drug_era only per oral during day -365 through -1 days relative to index: acetylcysteine | 0.023657181 |
| 81902102 | condition_occurrence during day -365 through -1 days relative to index: Urinary tract infectious disease | 0.021529295 |
| 43009000 | drug_era only per oral during day -365 through -1 days relative to index: etizolam | 0.021206806 |
| 4174764102 | condition_occurrence during day -365 through -1 days relative to index: Late gastric cancer | 0.020846421 |
| 4178818210 | condition_era group during day -365 through -1 days relative to index: Inflammation of specific body systems | 0.02021696 |
| 134736102 | condition_occurrence during day -365 through -1 days relative to index: Backache | 0.018635517 |
| 317585102 | condition_occurrence during day -365 through -1 days relative to index: Aortic aneurysm | 0.018265864 |
| 4313511210 | condition_era group during day -365 through -1 days relative to index: Neoplasm of uncertain behavior of intra-abdominal organs | 0.015482773 |
| 42873636 | drug_era only per oral during day -365 through -1 days relative to index: mirabegron | 0.011301574 |
| 4339468210 | condition_era group during day -365 through -1 days relative to index: Ear, nose and throat disorder | 0.010632856 |
| 937439 | drug_era only per oral during day -365 through -1 days relative to index: bethanechol | 0.010558223 |
| 442793210 | condition_era group during day -365 through -1 days relative to index: Complication due to diabetes mellitus | 0.009151262 |
| 4132130210 | condition_era group during day -365 through -1 days relative to index: Dilatation of aorta | 0.009135944 |
| 4051221210 | condition_era group during day -365 through -1 days relative to index: Increased lipid | 0.008981723 |
| 4294382502 | procedure_occurrence during day -365 through -1 days relative to index: Esophagogastroduodenoscopy | 0.006490527 |
| 4111798210 | condition_era group during day -365 through -1 days relative to index: Neoplasm of digestive organ | 0.005710341 |
| 4098954102 | condition_occurrence during day -365 through -1 days relative to index: Klatskin's tumor | 0.004803974 |
| 1002 | age in years | 0.004268913 |
| 4181351102 | condition_occurrence during day -365 through -1 days relative to index: Malignant tumor of ovary | 0.004165431 |
| 4098954210 | condition_era group during day -365 through -1 days relative to index: Klatskin's tumor | 0.002658715 |
| 19137056 | drug_era only per oral during day -365 through -1 days relative to index: theobromine | 0.002495356 |
| 3.01473E+12 | measurement value during day -365 through -1 days relative to index: Left ventricle [Length] Minor axis.diastole US.M-mode+Measured (millimeter) | 0.001865245 |
| 444208210 | condition_era group during day -365 through -1 days relative to index: Chronic inflammatory disorder | 0.000949482 |
| 42898675 | drug_era only per oral during day -365 through -1 days relative to index: Bacillus subtilis | 0.000781716 |
| 3.03628E+12 | measurement value during day -365 through -1 days relative to index: Body height (centimeter) | -0.004553607 |
| 4110575102 | condition_occurrence during day -365 through -1 days relative to index: Adenocarcinoma of rectum | -0.005992766 |
| 4.19359E+12 | measurement value during day -365 through -1 days relative to index: Peak expiratory flow rate before bronchodilation (liter per second) | -0.008314705 |
| 19058933 | drug_era only per oral during day -365 through -1 days relative to index: erdosteine | -0.008790957 |
| 43009008 | drug_era only per oral during day -365 through -1 days relative to index: rebamipide | -0.010134388 |
| 37109061502 | procedure_occurrence during day -365 through -1 days relative to index: CT angiography of brain and neck artery | -0.013953473 |
| 3.02202E+12 | measurement value during day -365 through -1 days relative to index: QRS duration (Unknown unit) | -0.014289142 |
| 443568210 | condition_era group during day -365 through -1 days relative to index: Malignant neoplasm of gastrointestinal tract | -0.015631409 |
| 4054503210 | condition_era group during day -365 through -1 days relative to index: Neoplasm of intra-abdominal organs | -0.015792106 |
| 723013 | drug_era only per oral during day -365 through -1 days relative to index: diazepam | -0.018581929 |
| 974166 | drug_era only per oral during day -365 through -1 days relative to index: hydrochlorothiazide | -0.018845588 |
| 4148972502 | procedure_occurrence during day -365 through -1 days relative to index: Extubation of trachea | -0.020628328 |
| 45775324 | drug_era only per oral during day -365 through -1 days relative to index: diatrizoic acid | -0.020663993 |
| 19017742 | drug_era only per oral during day -365 through -1 days relative to index: febuxostat | -0.026411591 |
| 40486678210 | condition_era group during day -365 through -1 days relative to index: Swelling of trunk | -0.027201534 |
| 4344497210 | condition_era group during day -365 through -1 days relative to index: Soft tissue lesion | -0.030973664 |
| 198124210 | condition_era group during day -365 through -1 days relative to index: Kidney disease | -0.033410697 |
| 1125315 | drug_era only per oral during day -365 through -1 days relative to index: acetaminophen | -0.034088425 |
| 4132552210 | condition_era group during day -365 through -1 days relative to index: Acute digestive system disorder | -0.038585785 |
| 437663102 | condition_occurrence during day -365 through -1 days relative to index: Fever | -0.041410431 |
| 4028367210 | condition_era group during day -365 through -1 days relative to index: Acute disease of cardiovascular system | -0.044027243 |
| 200219210 | condition_era group during day -365 through -1 days relative to index: Abdominal pain | -0.045470645 |
| 19011339 | drug_era only per oral during day -365 through -1 days relative to index: mosapride | -0.04690956 |
| 4235697802 | observation during day -365 through -1 days relative to index: Post-discharge follow-up | -0.048946131 |
| 4174763102 | condition_occurrence during day -365 through -1 days relative to index: Early gastric cancer | -0.052043496 |
| 19005512 | drug_era only per oral during day -365 through -1 days relative to index: trimebutine | -0.05570021 |
| 201820102 | condition_occurrence during day -365 through -1 days relative to index: Diabetes mellitus | -0.058644107 |
| 3.01373E+12 | measurement value during day -365 through -1 days relative to index: Hepatitis B virus surface Ab [Units/volume] in Serum or Plasma by Immunoassay (unit per liter) | -0.06164851 |
| 4130997210 | condition_era group during day -365 through -1 days relative to index: Neoplasm of ascending colon | -0.063037532 |
| 1103314 | drug_era only per oral during day -365 through -1 days relative to index: tramadol | -0.06801226 |
| 970250 | drug_era only per oral during day -365 through -1 days relative to index: spironolactone | -0.068195857 |
| 443258210 | condition_era group during day -365 through -1 days relative to index: Mass in head or neck | -0.072435473 |
| 378416210 | condition_era group during day -365 through -1 days relative to index: Retinal disorder | -0.078142364 |
| 4297400210 | condition_era group during day -365 through -1 days relative to index: Mild cognitive disorder | -0.083308455 |
| 316866210 | condition_era group during day -365 through -1 days relative to index: Hypertensive disorder | -0.08539318 |
| 443387102 | condition_occurrence during day -365 through -1 days relative to index: Malignant tumor of stomach | -0.086056845 |
| 1797513 | drug_era only per oral during day -365 through -1 days relative to index: ciprofloxacin | -0.087269588 |
| 201340210 | condition_era group during day -365 through -1 days relative to index: Gastritis | -0.095741198 |
| 939976 | drug_era only per oral during day -365 through -1 days relative to index: sodium sulfate | -0.105675284 |
| 77030102 | condition_occurrence during day -365 through -1 days relative to index: Disorder of breast | -0.106709135 |
| 1716903 | drug_era only per oral during day -365 through -1 days relative to index: moxifloxacin | -0.107556824 |
| 4104000210 | condition_era group during day -365 through -1 days relative to index: Lesion of liver | -0.115637055 |
| 4145825210 | condition_era group during day -365 through -1 days relative to index: Anorectal disorder | -0.117885608 |
| 4088035802 | observation during day -365 through -1 days relative to index: Screening status | -0.120023256 |
| 1367500 | drug_era only per oral during day -365 through -1 days relative to index: losartan | -0.12102755 |
| 3.01626E+12 | measurement value during day -365 through -1 days relative to index: Waist Circumference at umbilicus by Tape measure (centimeter) | -0.123847768 |
| 443390102 | condition_occurrence during day -365 through -1 days relative to index: Malignant tumor of rectum | -0.126392408 |
| 19112563 | drug_era only per oral during day -365 through -1 days relative to index: calcium polystyrene sulfonate product | -0.128388074 |
| 199860210 | condition_era group during day -365 through -1 days relative to index: Hernia of abdominal cavity | -0.128720821 |
| 4036803502 | procedure_occurrence during day -365 through -1 days relative to index: General examination of patient | -0.130975251 |
| 35608047502 | procedure_occurrence during day -365 through -1 days relative to index: CT of stomach with contrast | -0.137564309 |
| 4116228102 | condition_occurrence during day -365 through -1 days relative to index: Papillary thyroid carcinoma | -0.141652233 |
| 4043371210 | condition_era group during day -365 through -1 days relative to index: Inflammatory disorder of digestive tract | -0.143017185 |
| 4143250210 | condition_era group during day -365 through -1 days relative to index: Mass of colon | -0.144053419 |
| 4223938102 | condition_occurrence during day -365 through -1 days relative to index: Dizziness | -0.148090027 |
| 4182572802 | observation during day -365 through -1 days relative to index: Radiotherapy to abdomen | -0.150034998 |
| 78162210 | condition_era group during day -365 through -1 days relative to index: Peripheral vertigo | -0.152222732 |
| 443530102 | condition_occurrence during day -365 through -1 days relative to index: Hematochezia | -0.157218849 |
| 4246125210 | condition_era group during day -365 through -1 days relative to index: Primary malignant neoplasm of large intestine | -0.160906481 |
| 77670102 | condition_occurrence during day -365 through -1 days relative to index: Chest pain | -0.167610865 |
| 4.23304E+12 | measurement value during day -365 through -1 days relative to index: FEV1/FVC ratio before bronchodilator (percent) | -0.172684325 |
| 40486024210 | condition_era group during day -365 through -1 days relative to index: Cyst of abdomen | -0.195569084 |
| 734354 | drug_era only per oral during day -365 through -1 days relative to index: pregabalin | -0.195678596 |
| 4103192210 | condition_era group during day -365 through -1 days relative to index: Myocardial lesion | -0.196533759 |
| 4235749502 | procedure_occurrence during day -365 through -1 days relative to index: Laparoscopic-assisted anterior resection of rectum | -0.205690234 |
| 4256761210 | condition_era group during day -365 through -1 days relative to index: Imaging result abnormal | -0.219620779 |
| 4297400102 | condition_occurrence during day -365 through -1 days relative to index: Mild cognitive disorder | -0.219937664 |
| 1154332 | drug_era only per oral during day -365 through -1 days relative to index: pseudoephedrine | -0.222677768 |
| 939506 | drug_era only per oral during day -365 through -1 days relative to index: sodium bicarbonate | -0.225925083 |
| 4027369210 | condition_era group during day -365 through -1 days relative to index: Vascular disease of abdomen | -0.236453227 |
| 192279102 | condition_occurrence during day -365 through -1 days relative to index: Disorder of kidney due to diabetes mellitus | -0.242367149 |
| 4113547210 | condition_era group during day -365 through -1 days relative to index: Lesion of stomach | -0.242840483 |
| 3.02459E+12 | measurement value during day -365 through -1 days relative to index: FEV1/FVC Predicted (percent) | -0.245391132 |
| 966991 | drug_era only per oral during day -365 through -1 days relative to index: simethicone | -0.249458157 |
| 3.04311E+12 | measurement value during day -365 through -1 days relative to index: Platelet mean volume [Entitic volume] in Blood by Automated count (femtoliter) | -0.252509663 |
| 4174763210 | condition_era group during day -365 through -1 days relative to index: Early gastric cancer | -0.254087184 |
| 4304943502 | procedure_occurrence during day -365 through -1 days relative to index: Endoscopic retrograde cholangiopancreatography | -0.259896643 |
| 4134595210 | condition_era group during day -365 through -1 days relative to index: Chronic disease of genitourinary system | -0.265673523 |
| 135772210 | condition_era group during day -365 through -1 days relative to index: Goiter | -0.265903319 |
| 8532001 | gender = FEMALE | -0.270074006 |
| 4059290210 | condition_era group during day -365 through -1 days relative to index: Steatosis of liver | -0.2718368 |
| 46271022102 | condition_occurrence during day -365 through -1 days relative to index: Chronic kidney disease | -0.274509111 |
| 4109085210 | condition_era group during day -365 through -1 days relative to index: Right sided abdominal pain | -0.280221247 |
| 4091457802 | observation during day -365 through -1 days relative to index: Cardiac rhythm type | -0.288549871 |
| 438112210 | condition_era group during day -365 through -1 days relative to index: Neoplastic disease | -0.290630109 |
| 4216397210 | condition_era group during day -365 through -1 days relative to index: Nerve root disorder | -0.293662358 |
| 4178956210 | condition_era group during day -365 through -1 days relative to index: Vascular disorder of extremity | -0.297591625 |
| 4134294210 | condition_era group during day -365 through -1 days relative to index: Acute inflammatory disease | -0.302518881 |
| 4302836210 | condition_era group during day -365 through -1 days relative to index: Neoplasm of endocrine gland | -0.304006234 |
| 40488439210 | condition_era group during day -365 through -1 days relative to index: Abnormality of systemic vein | -0.304031011 |
| 198464102 | condition_occurrence during day -365 through -1 days relative to index: Incisional hernia | -0.35663044 |
| 197500210 | condition_era group during day -365 through -1 days relative to index: Primary malignant neoplasm of colon | -0.360268219 |
| 3.02581E+12 | measurement value during day -365 through -1 days relative to index: Q-T interval (Unknown unit) | -0.394270721 |
| 1510813 | drug_era only per oral during day -365 through -1 days relative to index: rosuvastatin | -0.44373333 |
| 45765544502 | procedure_occurrence during day -365 through -1 days relative to index: CT of thyroid with contrast | -0.55649427 |
| 4288544210 | condition_era group during day -365 through -1 days relative to index: Inguinal hernia | -0.593946097 |
| 3.03366E+12 | measurement value during day -365 through -1 days relative to index: Prothrombin time (PT) actual/Normal (percent) | -0.607576045 |
| 141253102 | condition_occurrence during day -365 through -1 days relative to index: Disorder of thyroid gland | -0.609852814 |
| 195856210 | condition_era group during day -365 through -1 days relative to index: Cholangitis | -0.708399219 |
| 4115576210 | condition_era group during day -365 through -1 days relative to index: Lesion of gallbladder | -0.762413918 |
| 4028373210 | condition_era group during day -365 through -1 days relative to index: Hernia of abdominal wall | -1.08497366 |
| 3.02456E+12 | measurement value during day -365 through -1 days relative to index: Albumin [Mass/volume] in Serum or Plasma (gram per deciliter) | -1.298990633 |
| 81251210 | condition_era group during day -365 through -1 days relative to index: Neoplasm of breast | -1.451477301 |
